# Anatomy of a viral entry platform differentially functionalized by integrins α3 and α6

**DOI:** 10.1038/s41598-020-62202-9

**Published:** 2020-03-24

**Authors:** Jérôme Finke, Snježana Mikuličić, Anna-Lena Loster, Alexander Gawlitza, Luise Florin, Thorsten Lang

**Affiliations:** 1 Department of Membrane Biochemistry, Life & Medical Sciences (LIMES) Institute, University of Bonn, Carl-Troll-Straße 31, 53115 Bonn, Germany; 2 Institute for Virology and Research Center for Immunotherapy (FZI), University Medical Center of the Johannes Gutenberg University Mainz, Obere Zahlbacher Straße 67, 55131 Mainz, Germany

**Keywords:** Human papilloma virus, Nanoscale biophysics, Mechanisms of disease

## Abstract

During cell invasion, human papillomaviruses use large CD151 patches on the cell surface. Here, we studied whether these patches are defined architectures with features for virus binding and/or internalization. Super-resolution microscopy reveals that the patches are assemblies of closely associated nanoclusters of CD151, integrin α3 and integrin α6. Integrin α6 is required for virus attachment and integrin α3 for endocytosis. We propose that CD151 organizes viral entry platforms with different types of integrin clusters for different functionalities. Since numerous viruses use tetraspanin patches, we speculate that this building principle is a blueprint for cell-surface architectures utilized by viral particles.

## Introduction

Tetraspanins are a family of small membrane proteins, primarily localized at the cell surface. They associate with one another and numerous partner proteins to form tetraspanin enriched microdomains (TEMs)^1^. TEMs are involved in a large variety of basic cellular processes and pathological mechanisms like pathogen entry^2^. For instance, tetraspanin CD151 mediates infections by human papillomavirus (HPV) and human cytomegalovirus^3^.

Apparently, CD151 has a crucial role in the early events of the HPV infection pathway on the cell surface. Here, HPV particles associate with large CD151 patches to which they remain bound to, until they co-internalize with them^4,5^. Additionally, HPVs associate with aggregates of other tetraspanins^3,6^ and several other viruses colocalize with tetraspanin patches or use tetraspanin assemblies for cell entry as well^7–9^. These observations lead to the proposal that virus contact with the cell membrane triggers the formation of tetraspanin-based virus entry platforms^6^. Such viral entry platforms would require at least two functionalities: attachment and internalization of the virus.

Tetraspanins could have a role in organizing TEMs by recruiting non-tetraspanin components for virus binding and internalization. Candidates comprise members of the integrin family of cell-adhesion-receptors that (i) interact with CD151, (ii) have a connection to HPV infection, and (iii) are integral part of TEMs. Integrin α6 is a strong candidate as it interacts directly with CD151^10^ and, among others, is one of the direct receptors for HPV binding^11–13^. Hence, integrin α6 may be organized into TEMs for virus binding. Another CD151 interacting integrin is integrin α3^10^. Its role in infection is less clear as it is required for HPV infection of HeLa^5^ but not of HaCaT cells^14^.

While integrin α6 mediates virus binding directly, other integrins could be involved indirectly by recruiting other well-established primary receptors such as heparan sulfate proteoglycans (HSPGs)^15–18^. Alternatively, integrins could mediate outside-in activation of signalling pathways^19^, which could link external virus binding to intracellular processes like endocytosis^20^.

Similar to CD151, integrin α6 and perhaps also integrin α3 are implicated in the infection process of HPV16; it is tempting to speculate that these proteins assemble on the cell-surface to form a viral entry platform. To shed light on this question, we tested the effect of integrin knockdown on infection in a luciferase-based infection assay. Moreover, we studied intracellular virus processing and cell-surface binding by Western blot analysis and microscopy. Finally, we examined the organization of integrin α3, integrin α6 and CD151 by super-resolution microscopy. We found out that both integrins play a role in infection and cellular uptake, but only integrin α6 is required for cell-surface binding. In addition to this, the local density of integrin α3, integrin α6 and CD151 cluster increases at viral particle attachment sites. Our data suggests that viral particles use an entry platform containing integrin α6 for virus attachment and integrin α3 for internalisation.

## Results

### Integrin α3 is required for HPV16 infection of keratinocytes

**Figure 1:**
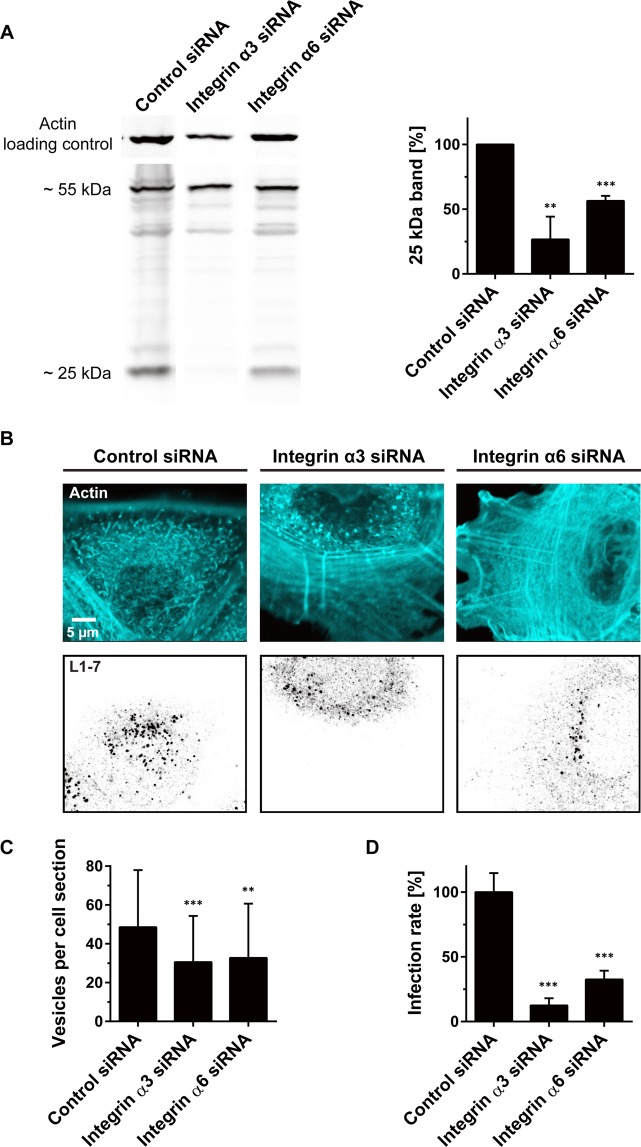
*Integrin α3 or integrin α6 knockdown inhibits viral capsid processing and infection*. Integrin α3 or integrin α6 were knocked down in HaCaT cells by siRNA transfection (for knockdown efficiency see Fig. S1). (**A**) Two days after transfection, cells were incubated for 24 h with HPV16 PsVs, washed, lysed and analyzed by Western blot for the viral protein L1 and its ~25 kDa cleavage product. For clarity, lanes were cropped from original blots shown in full in Fig. S14 (L1) and S15 (actin). Values were related to the control which was set to 100% and are given as means ± SD (n = 3 independent experiments). (**B**,**C**) Two days after transfection, cells were incubated for 5 h with HPV16 PsVs, washed fixed, stained with an antibody that recognizes L1 after capsid disassembly, and imaged by confocal microscopy taking an optical section from the cell body. (**B**) Actin (cyan) and L1–7 (inverted greyscale) each are displayed at the same arbitrary scaling (linear lookup tables). From the optical section (**B**), an image analysis algorithm counted the number of detected vesicles per cell (**C**) and quantified the vesicle staining intensity (Fig. S2). Values are given as means ± SD (n = 60 analysed cells collected from three biological replicates). (**D**) HaCaT cells were transfected and incubated with PsVs as in (**A**) with the difference that on the encapsidated plasmid luciferase expression is under the control of the HPV16 promoter instead of the CMV promoter. One day after adding PsVs, cells were lysed and the infection rate was assessed by analysing the luciferase activity. For normalization to cell number, luciferase activity was related to the dehydrogenase activity. Values are expressed as percent of control (average of control was set to 100%). Values are given as means ± SD (n = 20–21 technical replicates collected from five biological replicates). Unpaired Student’s t-test, comparing control to knockdown conditions (***p < 0.001; **p < 0.01).

To clarify the role of integrin α3 in the infection of keratinocytes, we infected integrin α3 depleted HaCaT cells by incubation with HPV16 pseudovirions (PsVs). For positive control, cells were depleted from integrin α6, which is required for infection of HeLa and KH-SV cells^5,21^. In Western blot analysis, knockdown reduced integrin α6 and integrin α3 protein levels by 87% and 97%, respectively (Fig. S1). Intracellular processing of the L1 virus capsid protein was monitored by Western blot analysis and by microscopy. In the Western blots, we quantified the ~25 kDa cleavage product of L1, which is generated only after internalization and lysosomal degradation of the virus^22^. As seen in Fig. 1A, knockdown of integrin α3 and integrin α6 reduced the cleavage product by 73% and 44%, respectively. For microscopy, we employed antibody-detection of the L1–7 epitope, which only becomes detectable after intracellular capsid disassembly^4,23^. Knockdown of either one of the integrins reduced the number of L1–7 positive organelles to about 65% (Fig. 1B,C). Moreover, organelles were slightly dimmer (Fig. S2), suggesting that apart from less forming endocytic organelles the viral load per organelle diminishes.

**Figure 2:**
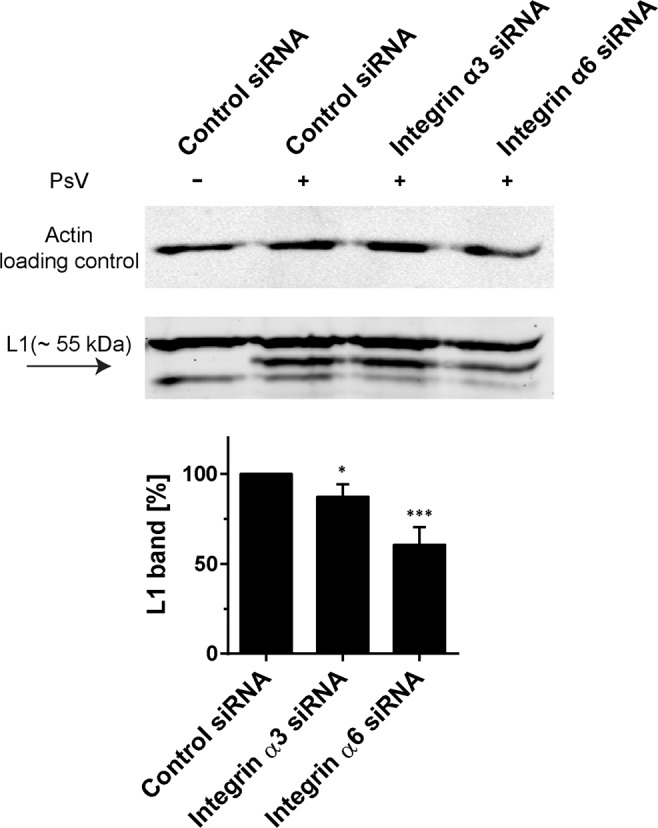
Knockdown of integrin α6 but not of integrin α3 strongly inhibits PsV binding. Two days after siRNA transfection, cells were incubated for 1 h at 0 °C with HPV16 PsVs, scraped from the substrate, washed, lysed and analyzed by Western blot. Full Western blots are shown in Fig. S16 (L1) and S17 (actin). Binding of viral particles was assayed by quantification of the L1 ~55 kDa band. The control value was set to 100% and used for normalization. Values are given as means ± SD (n = 4 independent experiments). Unpaired Student’s t-test, comparing control to knockdown conditions (***p < 0.001; *p < 0.05).

Next, we employed a luciferase-based infection assay to test whether less uptake and processing of the L1 protein would be associated with a lower infection rate. Infection is inhibited by 88% and 67% after integrin α3 and integrin α6 knockdown, respectively (Fig. 1D). It should be noted that integrin knockdown has secondary effects (Fig. S3). Still, after correcting for these secondary effects, knockdown of each integrin more than halves the infection rate (Fig. S3). These experiments demonstrate that apart from integrin α6, integrin α3 plays a role in HPV infection of HaCaT cells as well. However, in Western blot analysis testing PsVs cell-surface primary binding only the knockdown of integrin α6 strongly inhibits binding by 40% (Fig. 2), suggesting each of these integrins is differently involved during infection.

### CD151/integrin distribution

Biochemical pulldown assays suggest that integrin α3 and integrin α6 directly interact with CD151^24–26^. Consequently, these molecules should co-localize in the cell membrane. For verification, we studied triple co-localization of GFP-labelled CD151, integrin α3 and integrin α6 in the cell membrane. We employed cell-free membrane sheets to assure that we examine only plasma membrane associated proteins. Furthermore, STED super-resolution microscopy was employed, which enabled us to resolve diffraction limited signals into smaller spots (Fig. S4).

**Figure 3:**
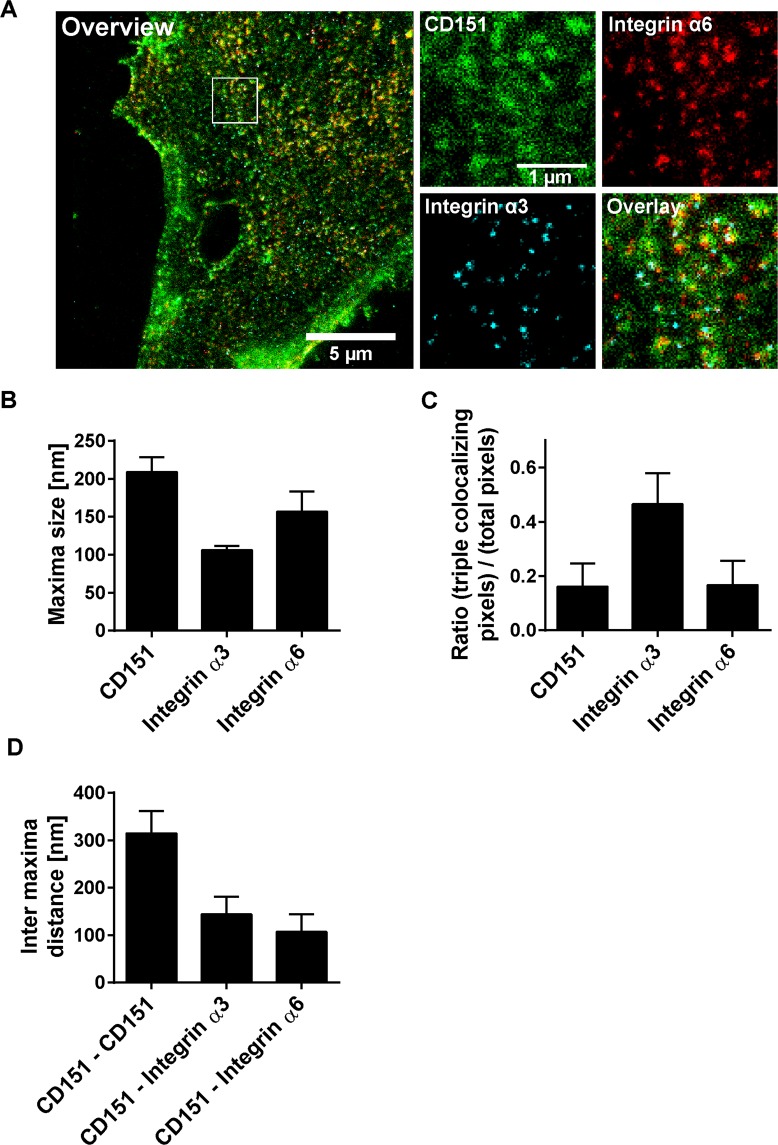
*Characteristics of CD151 and integrin maxima*. CD151-GFP was transfected into HaCaT cells. CD151-GFP and endogenous CD151 localize to the same domains (Fig. S7E,F) and CD151-GFP is as functional as non-tagged CD151 (Fig. S8). One day after transfection, cells were treated for 5 h without or with PsVs (Fig. S9), washed, membrane sheets were generated, stained and analyzed by three channel STED microscopy. Green lookup table, CD151-GFP visualized by nanobodies; red and cyan lookup tables, integrin α6 and integrin α3 stained by antibody labeling, respectively. Images are displayed at arbitrary intensity scalings (linear lookup tables). Staining with the integrin antibodies is highly specific (Fig. S1). (**A**) Large panel, membrane sheet (channel overlay). The white box marks an area from which magnified views of the individual channels are shown. From regions of interest (ROIs) we measured (**B**) maxima size and signal overlap (**C**). For (**C**), for all three channels within a ROI the pixels with an intensity higher than the average ROI intensity were selected. Then, the number of pixels positive in all three channels were related to the number of all positive pixels in one specific channel as indicated. (**D**) Shortest inter-maxima distances of CD151-GFP to CD151-GFP, integrin α3 or integrin α6. Values are given as means ± SD (n = 60 membrane sheets collected from three biological replicates).

In the case of integrin α3 and integrin α6, the majority of the intensity maxima in the cell membrane are well-defined spherical structures with an average diameter of 106 nm and 157 nm, respectively (Fig. 3A,B). The sizes show a lognormal distribution, in particular for integrin α3 (Fig. S5). After correcting for the blurring by the point spread function (PSF) of the microscope, we obtained a size of 84 nm and 123 nm for integrin α3 and integrin α6 maxima (Fig. S6), respectively. Integrin α3 maxima are less numerous, likely because its expression level is lower when compared to integrin α6^27^. CD151-GFP maxima are larger than 200 nm and less-defined, with more background signal in their neighbourhood (Fig. 3A). Correction for the PSF yields a diameter of 189 nm. The lower signal-to-noise ratio is due to the lower signal intensity of the nanobody staining applied for the visualization of CD151-GFP in STED microscopy. CD151-GFP maxima size and density depend on the expression level similar to endogenous CD151 maxima. Moreover, CD151-GFP and endogenous CD151 occupy the same domains (Fig. S7).

Studying the relationship between the different protein maxima, we find that a high fraction of integrin α3-signal overlaps with integrin α6- and CD151-GFP-signals (Fig. 3C). The overlap between CD151-GFP or integrin α6 with the respective other two components is lower (Fig. 3C). The average distance of a CD151-GFP maxima-centre to the centre of its nearest integrin α3 or integrin α6 maxima is 144 and 107 nm, respectively (Fig. 3D). The distances are in the range of the maxima sizes which suggests that the maxima do not perfectly overlap but are closely associated.

### PsVs associate with cluster crowds

**Figure 4:**
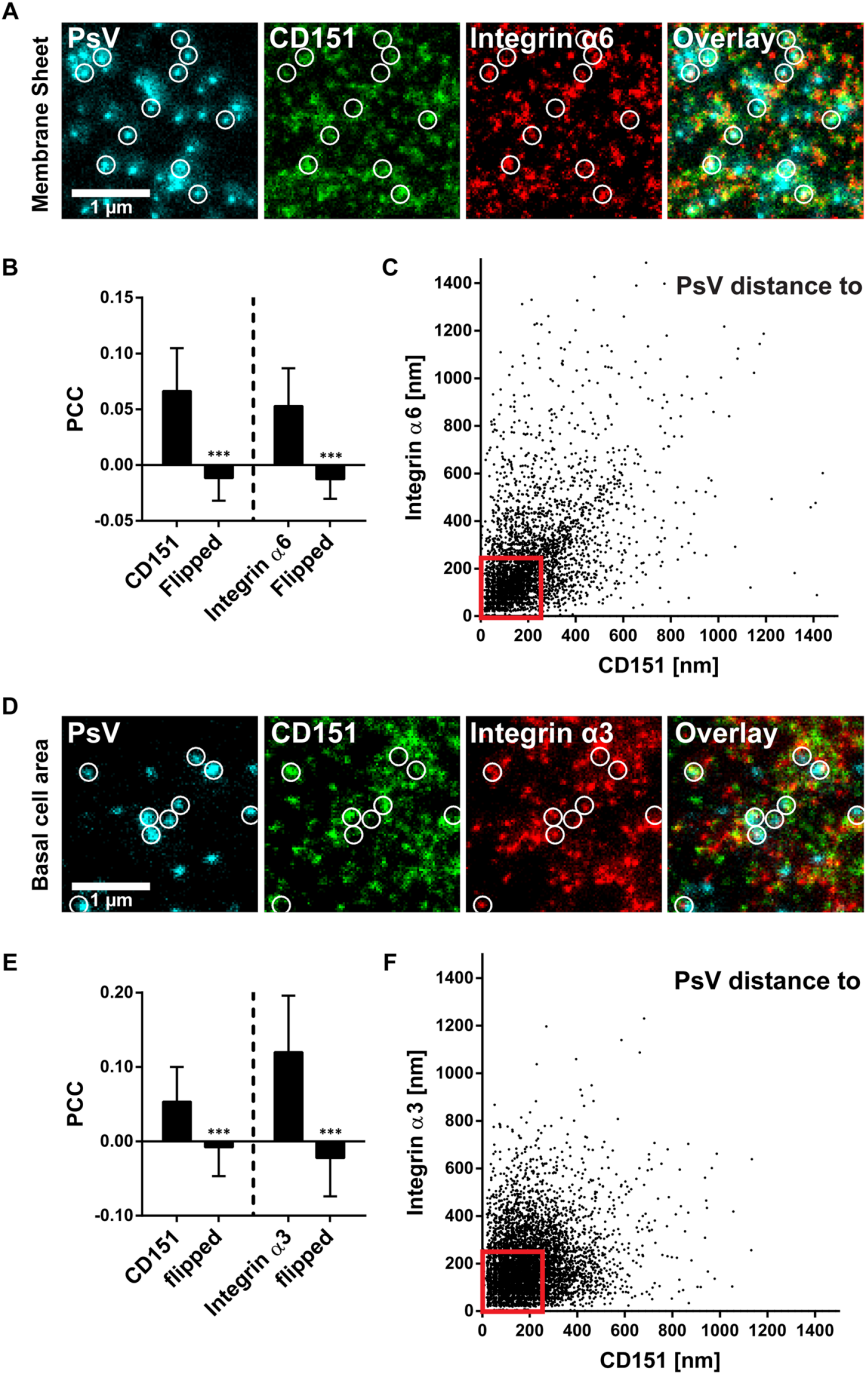
*Viral particle distance to CD151 and integrin maxima*. (**A**–**C**) Membrane sheets were generated from HaCaT cells treated for 5 h with EdU-PsVs. They were stained for EdU-PsVs (cyan lookup table; click-labelled with fluorescein), CD151 (green lookup table; immunostaining) and integrin α6 (red lookup table; immunostaining), followed by STED microscopy imaging. (**A**) Images from the three channels and an overlay are shown and displayed at arbitrary intensity scalings (linear lookup tables). Circles mark identical pixel locations. (**B**) Pearson correlation coefficient between PsVs and CD151 (left) and integrin α6 (right). A control value for randomized distribution (‘flipped’) was calculated after flipping of one channel. (**C**) For individual PsVs, we plotted their nearest distance to a CD151 and an integrin α6 maximum (the analysis includes 4452 PsVs from 57 membrane sheets collected from three biological replicates). (**D**–**F**) As in (**A**–**C**) but cells were analysed and we stained for integrin α3 (the plot in F includes 8519 analyzed PsVs from 60 cells collected from three biological replicates). The pattern of PsV binding to the cell membrane looks the same on cells and membrane sheets (Fig. S10). Values are given as means ± SD (n = 57 membrane sheets or 60 cells, each collected from three biological replicates). Statistical analysis was performed employing the unpaired Student’s t-test comparing the original to the flipped images (***p < 0.001). Red boxes frame viral particles potentially used for further analysis of the platform area (Fig. 5). Only PsVs were considered that were closer than 250 nm to bright CD151/integrin maxima.

Next, we asked whether PsV particles are closer to their potential binding partner integrin α6, and analysed the distances between PsVs and CD151/integrin maxima. On membrane sheets,  PsVs are often close to or overlap with CD151 or integrin α6 maxima (Fig. 4A,B). Most distances are in the range of several 100 nm (Fig. 4C), with no trend towards shorter distances to integrin α6 maxima. Also, no maxima preference is observed on PsVs/CD151/integrin α3 stainings of cells (Fig. 4D–F), or PsVs/CD151-GFP/integrin α3 stainings on membrane sheets (Fig. S11). Moreover, it should be noted that PsVs diminish the level of CD151 and integrins by 10–22% (Fig. S9).

**Figure 5:**
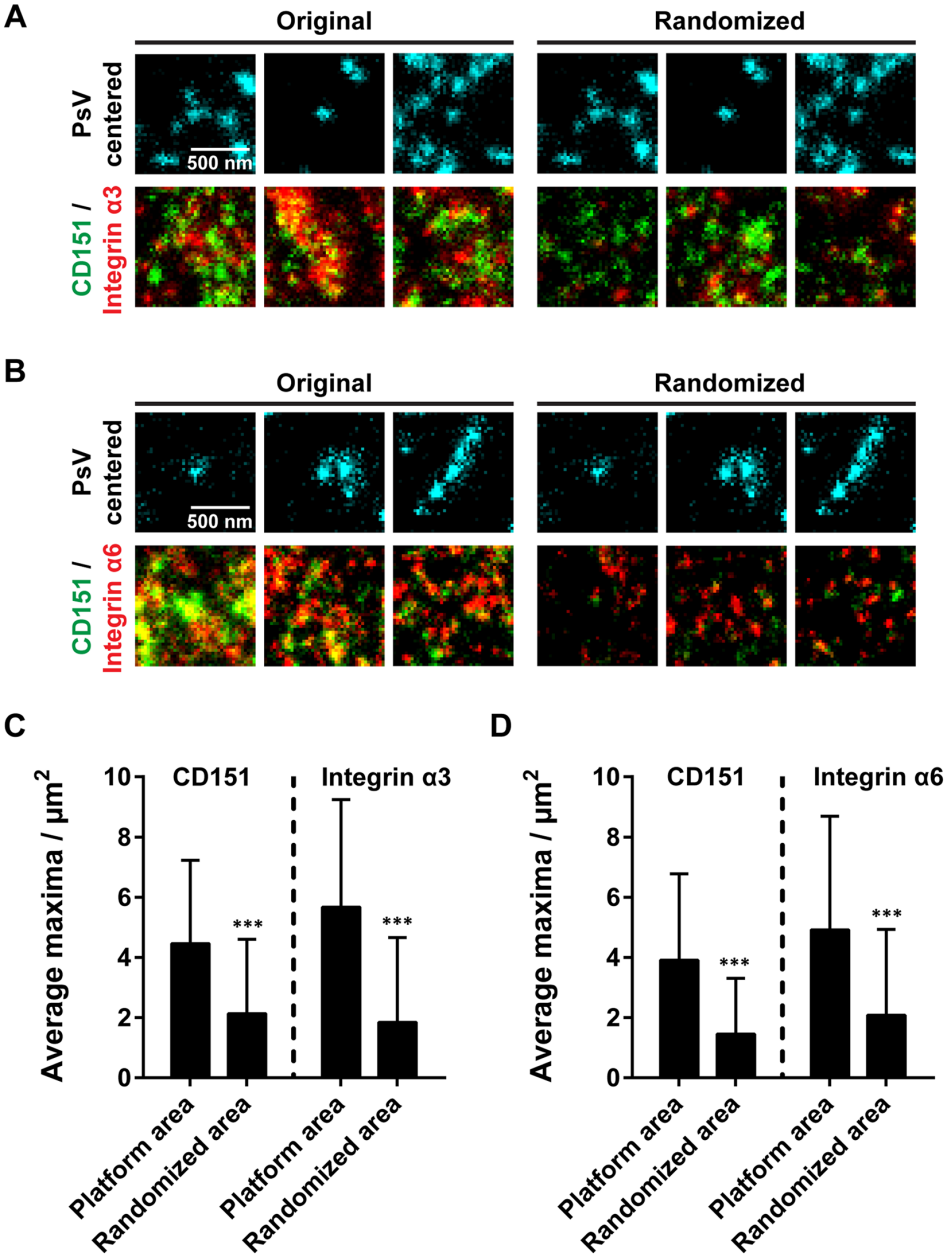
*Local crowding of clusters at virus attachment sites*. To characterize the anatomy of a possibly forming viral platform area, we selected those PsVs from Fig. 4C,F with a distance smaller than 250 nm to both a bright CD151 and integrin maximum (for examples see centrally located PsVs in the left panels in A and B). In a PsV-centred 1 µm × 1 µm ROI the number of bright maxima (see methods) in the CD151 and integrin channels were counted. The same was performed after flipping the CD151/integrin channels, causing a randomized relationship between PsV-attachment sites and maxima (A and B, right panels). In the original images more closely associated maxima are present at sites of PsV-attachment. Images are displayed in cyan (PsV) green (CD151) and red (integrin) at arbitrary intensity scalings (linear lookup tables). (**C**,**D**), quantification of the bright maxima in the 1 µm × 1 µm ROI. Values are given as means ± SD (n = 678 (**C**) and 283 (**D**) 1 µm × 1 µm ROIs, respectively). Statistical analysis was performed employing the unpaired Student’s t-test comparing the original to the randomized condition (***p < 0.001). Please note, that the number of clusters per attachment site was highly variable. For a histogram illustrating the variability see Fig. S12.

Are viral particles associated with a special type of membrane area containing the studied proteins enriched in protein clusters? We studied the local maxima composition at PsV-attachment sites in those  PsVs/CD151/integrin triple stainings. As not all maxima may arise from protein clusters but single molecules, we only included the ~10–20% brightest maxima. PsVs were considered to be possibly attached to CD151/integrins when they were closer than 250 nm to the next CD151 and integrin cluster. Then, we counted clusters within a 1 µm × 1 µm area centred to the PsV position. We found an average of 4–5 clusters of each type (Fig. 5). On the level of individual attachment sites, the values are highly variable, ranging from only one CD151 and integrin α6 cluster to nine CD151 and 22 integrin α6 clusters (Fig. S12). In contrast, in a randomly chosen location only  about 2 clusters of each type are present (Fig. 5). This suggests that PsVs associate with a specialized type of membrane area characterized by crowded CD151 and integrin clusters, and that this membrane area is utilized as an entry platform.

Apart from the crowding effect, we cannot recognize a defined shape, size or composition of the platform area – hence, the entry platform as a whole. Altogether, focusing on the brightest maxima, we find that the density of CD151 and integrin clusters is increased in the viral platform area.

### Integrin α6 has a role in viral particle binding and integrin α3 is required for internalization

**Figure 6:**
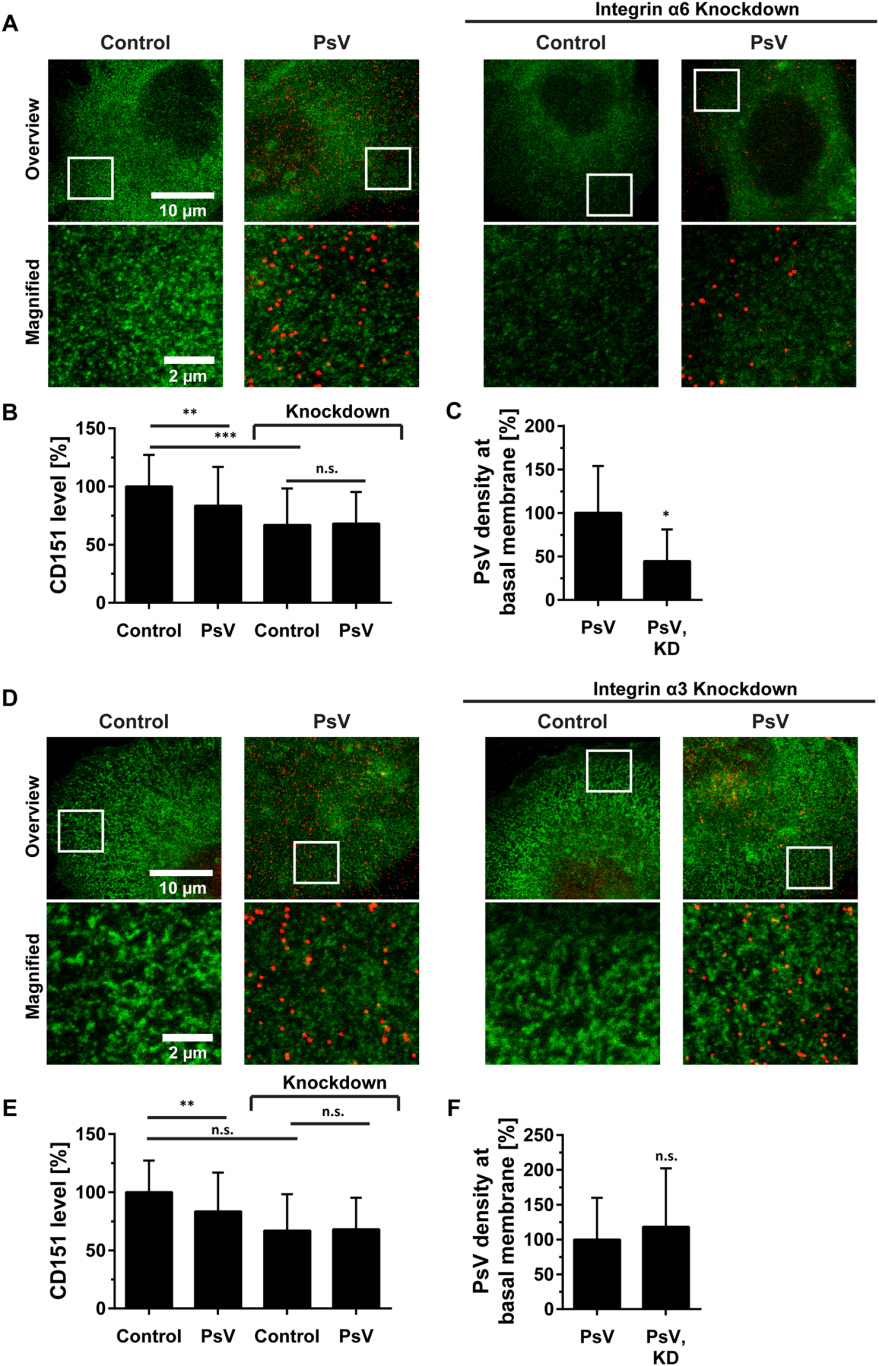
*Integrin α3 and integrin α6 knockdown differentially affect the CD151 level of the cell membrane and PsV-binding*. HaCaT cells were transfected with siRNA and two days later incubated without or with HPV16 PsVs for 5 h. Cells were fixed, permeabilized, immunostained for CD151 (green) and L1 (red), and imaged by STED microscopy. Images are displayed employing linear lookup tables. For A and D different scalings were applied, although all panels in A or D have the same arbitrary scaling. (**A**) Integrin α6 knockdown. For each condition, overlays from an overview and a magnified view are shown. (**B**) Average CD151 immunostaining intensity and (**C**) the PsVs particle density (100% correspond to 1.4 particles/μm^2^). (**D**–**F**) As (**A**–**C**), showing the integrin α3 knockdown (100% PsV density correspond to 1.2 particles/μm^2^). Values are shown as means ± SD (n = 60 cells collected from three biological replicates). The unpaired Student’s t-tests compare the conditions as indicated by the bars (**B**,**E**) or PsV to PsV and knockdown (**C**,**F**) (***p < 0.001; **p < 0.01; *p < 0.05).

In control cells, PsVs reduce the CD151 level by ≈20% (Fig. 6B), confirming the idea that CD151 in the basal membrane is reduced upon PsV incubation^5,28^. In the integrin α6  knockdown, PsV-binding is decreased by ≈55% (Fig. 6C). As less PsVs bind, the PsV-triggered reduction of cell-surface CD151 is abolished in the knockdown condition (Fig. 6B). It should be noted that in the knockdown less PsV binding and abolished PsV-triggered CD151 reduction occur on a lower CD151 level, since integrin α6 knockdown reduces the level of cell surface CD151 to ≈67% (Fig. 6B).

Integrin α3 knockdown does not reduce the CD151 cell-surface level (Fig. 6E) or the amount of PsVs bound to the cell surface (Fig. 6F). However, it diminishes the PsV-induced reduction of CD151 (Fig. 6E). As PsV binding is not affected, diminished CD151 internalization after knockdown suggests a role of integrin α3 in endocytosis.

### CD151 patches overlap with actin accumulations

**Figure 7:**
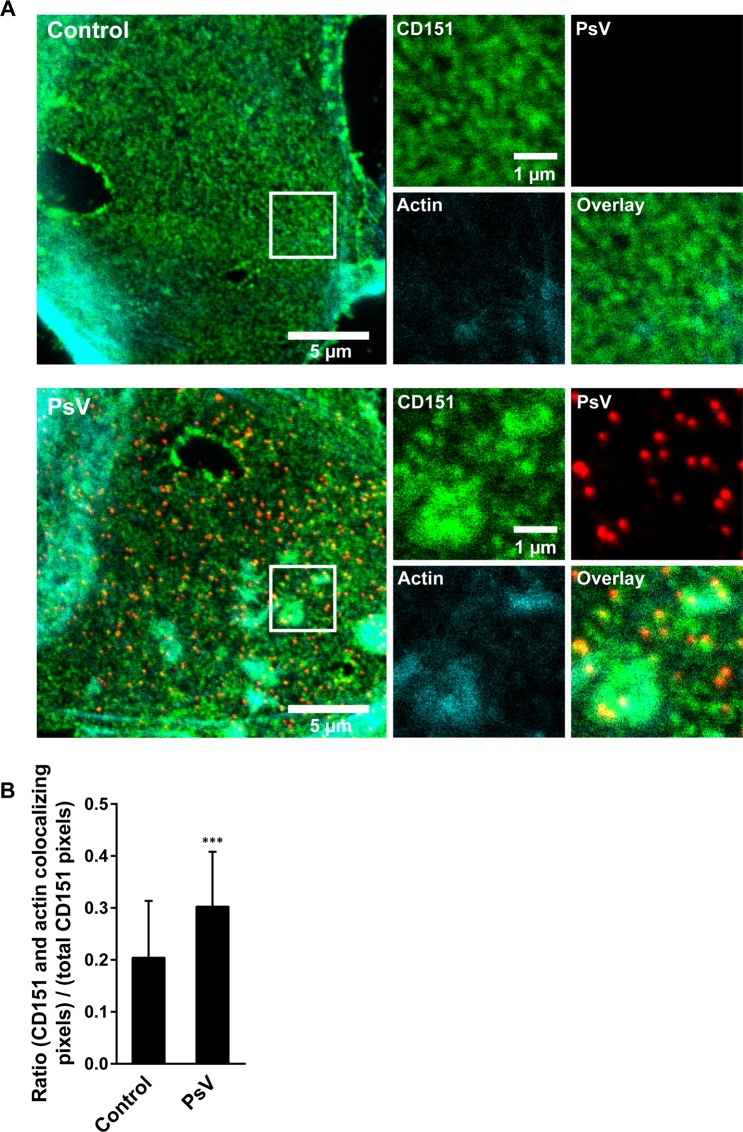
*Large CD151 patches coincide with intracellular actin accumulations*. CD151-GFP transfected HaCaT cells were treated for 3 h without or with PsVs. Membrane sheets were generated, stained, and imaged by confocal microscopy. Green (CD151-GFP; GFP signal was enhanced by nanobodies), red (PsVs visualized by L1 antibody labeling) and cyan (filamentous actin; fluorescently labelled phalloidin). Images are displayed using a linear lookup table. For each channel, the same arbitrary scaling was applied. (**A**) For each condition a membrane sheet is shown. Magnified views from the white boxes are shown, illustrating the individual channels. (**B**) For the CD151 and the actin channels, within a freehand ROI excluding membrane edges, the pixels with an intensity higher than the average ROI intensity were selected. Then, the number of pixels positive in both channels were related to the number of all positive pixels in the CD151 channel. Values are given as means ± SD (n = 45 membrane sheets collected from three biological replicates). Unpaired Student’s t-test (***p < 0.001).

As shown in Fig. 5, viral particles associate with CD151 cluster crowds (see also Fig. 7A and magnified view in Fig. S9), and may therefore co-internalize with several CD151 clusters. If PsVs concentrate CD151 clusters, and concentration is followed by actin accumulation at cluster crowds, PsVs should increase the overlap between CD151 and actin. In fact, we find an increase in overlap by ≈50% in the presence of PsVs (Fig. 7B).

## Discussion

### CD151/integrin distribution

Here, we studied the organization of CD151, integrin α3 and integrin α6 in the cell membrane. None of the three protein types is evenly distributed but rather concentrates in segregated maxima which have a diameter in the 100–200 nm range (Fig. 3B and S5). Determination of the exact size is difficult as the lognormal distribution of the maxima sizes (Fig. S5) suggests that our STED microscope cannot resolve size differences within the fraction of smaller maxima. Hence, as smaller maxima appear larger, we overestimate the average size. On the other hand, the smallest maxima may arise from single proteins and therefore are not protein clusters anyway. In any case, the data suggests that most maxima do not arise from single molecules but from larger aggregates as protein clusters, that typically are in the same size range as protein clusters formed by other proteins^29–31^.

However, the more relevant parameter is the distance between the maxima, as it allows differentiating between evenly mixed components in the same biological structure and closely associated but not mutually penetrating maxima. In the first case the maxima distance would be zero, in the latter case it is the sum of the radii of the two associated maxima. In this study, the inter-maxima distances range from 100–300 nm (Fig. 3D) and clearly point to closely associated maxima (see magnified view of the overlay in Fig. 3A). It should be noted that in this experiment CD151 was overexpressed. CD151-GFP and endogenous CD151 maxima depend in the same fashion from the expression level (Fig. S7A–D); overexpression increases the number of maxima, accompanied by a moderate increase in maxima size. On the other hand, the maxima density is underestimated due to the low-signal-to noise ratio of the nanobody (please note that the maxima density of CD151-GFP is lower than for endogenous CD151 (Fig. S7B,D)). This does not affect the CD151-integrin distances (107–144 nm; Fig. 3D) but leads to an overestimation of the CD151-CD151 inter-maxima distances (314 nm; Fig. 3D). Despite of this overestimation, we can safely conclude that the CD151 and integrin maxima do not perfectly overlap but are very close to one another. In other words, the three maxima types group together.

### Association of PsVs with CD151/integrin clusters

Viral particles associate with large cluster crowds (Fig. 5) which they may induce. The induction of large cluster crowds by viral particles is suggested by previous studies which employed tetraspanin overexpression^3,32^. It is possible that overexpression of tetraspanins promotes the growth of the tetraspanin crowds (see below), amplifying an otherwise moderate viral particle patching effect. In any case, our data would be also in line with a preference for associating with larger cluster crowds instead of inducing them. Altogether, we suggest that these crowds are entry platforms with a composition different from the rest of the cell membrane. On the level of individual PSV attachment sites, platform size and composition are highly variable (Fig. S12), perhaps because the analysis covers a full range of states from early to fully formed platforms. We speculate that a fully formed platform, briefly before internalization, is best represented by the example mentioned  above with at least 9 + 22 = 31 clusters (not including integrin α3) in an area of 1 µm^2^.

HPV dynamics at the cell membrane are complex, slow and unsynchronized^4,33–37^; however, they end with HPV internalisation. Earlier TIRF microscopy live imaging of plasma membrane events exemplified how only viral particles which are associated with a large CD151 aggregate internalize (here the aggregates were not resolved into smaller clusters). Moreover, upon internalisation, viral particles and CD151 signals both disappear from the TIRF field, indicating their co-internalisation^5^. In our experiments PsVs diminish the level of CD151-GFP by 22% (Fig. S9; see also Fig. 6B for a 20% diminshment of endogenous CD151), integrins diminish less. Effects are small because of (i) systematic underestimation (also uninfected cells are included in the analysis) and (ii) maxima are much more numerous than virus particles, so that viral particles can affect only a small maxima subpopulation. However, the data suggest that these components and PsVs are co-internalized, in line with the above-mentioned live imaging experiment.

Moreover, these earlier TIRF imaging experiments demonstrated that several hours after virus addition, about 80% of virus particles that are associated with large CD151 accumulations were internalized within a time frame of 2 to 4 minutes. Together with the findings of this study, the data indicate that virus particles are rapidly internalized after formation of the mature entry platform. Consequently, we likely see only very few fully formed platforms.

Attached PsVs are not closer to integrin α6 than to other maxima (Fig. 4). Perhaps this is because of clusters being reservoirs of active molecules^38^, representing only the non-interacting protein pool. In this case, PsVs could still be directly bound to integrin α6, although not to clusters but to single molecules released from the clusters into the platform area. Clusters being reservoirs would also explain the absence of perfect overlap between CD151- and integrin-maxima, as only molecules at the cluster peripheries interact. Alternatively, PsV binding to its receptor can decrease the accessibility of the antibody, which would also lead to a lack of signal overlap. However, it is possible that viral particles bind to another type of primary receptor in the platform, most likely HSPGs^36,39^, which would explain why half of the viral particles are still bound to the cells after knockdown, despite of a relatively high knockdown efficiency. Certainly, there must be more proteins in the platform, as for instance the tetraspanin CD63, which after PsV-contact with the cell-surface forms very similar patches or cluster crowds^3,4^. Other candidates are growth factor receptors which associate with CD151 and HPV16^28,40^. Moreover, integrins are present as heterodimers, possibly associating with molecules that couple integrin signalling to the cytoskeleton. Perhaps virus associated CD151 patches overlapping with actin accumulations (Fig. 7) are focal adhesion related signalling hubs linking intracellular actin bundles to extracellular components^41^. In line with this idea is focal adhesion kinase (FAK) being activated during HPV infection^21^.

CD151 likely has a role in the recruitment of integrins into TEMs^42–45^. The association with integrins is crucial, as CD151 mutants incapable of binding to integrins are unable to recover susceptibility to infection in CD151 depleted cells^5^. Additionally, CD151 may be necessary due to its requirement for co-transport of integrin α6 to the cell surface. CD151-integrin association occurs early during biosynthesis^46^, notably also with integrin α6 precursor forms^47^. Hence, co-transport of integrin α6 and CD151 from the ER to the cell membrane can explain the reduction in CD151 after integrin α6 knockdown (Fig. 6B). Mutually, integrin α6 increases upon overexpression of CD151-GFP (Fig. S13), supporting the idea of co-transport.

### Roles of integrin α6 and integrin α3

Studies employing co-immunoprecipitation and flow cytometry indicate that HPV binds directly to integrin α6^11–13^. On the other hand, it is also assumed that the  HPV capsid protein  binds to a set of primary receptors different from integrin α6^16,39^, undergoes conformational changes and is then transferred to a secondary receptor complex^34,48–50^ which could be part of a TEM. Our experiments do not allow to differentiate between these binding states which may coexist in the same cell membrane. Integrin α6 knockdown diminishing PsV-binding to 45% (Fig. 6C) confirms one substantial thing: integrin α6 is an important factor for virus attachment; nevertheless, the remaining 45% could be viral particles bound to the ECM or HSPGs acting in HaCaT cells as primary attachment sites^16,51^, or to additional components such as growth factor receptors^40^.

Integrin α3 is not necessary for virus binding, as PsV binding to the cell surface after knockdown was reduced poorly in our Western blot analysis, in contrast to integrin α6 (Fig. 2). Moreover, in microscopy, knockdown does not affect the PsVs detected at the basal cell membrane (Fig. 6F). However, integrin α3 is required for endocytosis as its depletion reduces all virus post-binding steps such as the PsVs induced reduction of the CD151 cell-surface level (Fig. 6E), L1 cleavage (Fig. 1A), capsid disassembly (Fig. 1C) and infection rate (Fig. 1D).

A role of integrin α3 in HaCaT cell infection contrasts a study by Aksoy *et al*. in which integrin α3 depletion had no effect^14^. However, this may be due to a lower multiplicity of infection and a different reporter system for infectivity in the mentioned study, which was a GFP reporter system in combination with flow cytometry.

Integrins are important players in signal transduction during HPV infection^21,52,53^ and integrin α3 could be an indirect effector. Integrin signalling (phosphoinositide-3-OH kinase (PI3K), FAK) is activated during HPV infection^21,53^. Interestingly, FAK is required for HPV uptake to early endosomes^21^ and integrin αv mediated internalization of adenovirus requires PI3K^54^. Integrin signalling could trigger intracellular actin dynamics^55–57^ which engulf the membrane. Hence, by binding simultaneously to several clusters, viruses may induce platform formation, actin reorganization, cell signalling, and finally endocytosis.

In a recent study, integrin α3 was shown to be directly involved in EGFR signalling^58^; this can be connected to a report showing that ADAM17-mediated EGFR signalling is involved in HPV entry platform formation and infection. Here, cell surface CD151 association with viral particles was dependent on EGFR mediated signalling^28^. Hence, integrin α3 may be required to sustain EGFR signalling as a prerequisite for HPV internalization. Additionally, integrin α3 signalling also seems to enhance the viral promoter activity (Fig. S3). Therefore, integrin α3 could influence signal transduction events not only for endocytosis, but for viral gene expression as well.

### Platforms and actin

The platform areas are sites of intracellular actin accumulation (Fig. 7). It is tempting to speculate that their maturation is accompanied by the intracellular accumulation of actin which is followed by endocytosis of the platform.

Our study explains a finding by Shigeta *et al*., where incubation of epidermal cells (A431) with anti-CD151 antibodies results in patched CD151-GFP co-localizing with actin^57^. Interestingly, treatment with anti-integrin α3 antibody had the very same effect, suggesting each antibody crosslinks the same cellular entity. This supports the idea of viral entry platform components defined by these molecules. Moreover, crosslinking of proteins from the extracellular site by antibodies or viral particles may be sufficient for triggering intracellular actin accumulation.

CD151 is involved in cytoskeletal reorganization^57^ and HPV internalization is an actin-dependent process^35,59^. We speculate that virus entry requires a considerable amount of CD151 clusters because for internalization actin-reorganization must be coordinated over a larger membrane area. The growth size of the platform may depend on the density of CD151 or other tetraspanin clusters and could therefore differ between cell types, and be influenced by overexpression as well. In the example shown in Fig. 7 CD151-GFP is overexpressed and the shown patches are larger than the platforms in Fig. 5, possibly due to an increased density of CD151 clusters which could have a promoting effect on the process of patching.

## Conclusion

To date, we are just at the beginning of understanding viral entry platforms. Actually, we only know that on the cell surface viruses use large patches of tetraspanins for entry. This study reveals the nano-architecture and functionalization of these platforms. We propose a model of a tetraspanin-organized viral entry platform with integrin α6 for virus attachment and integrin α3 for endocytosis. TEMs were shown to organize entry or budding sites for hepatitis C virus, coronavirus, influenza A virus and HIV, suggesting such platforms are widespread in viral infections^2,6,60^.

## Materials and Methods

### Antibodies and plasmid

Three HPV16 specific antibodies, a rabbit polyclonal antibody K75 (diluted 1:1000 for immunofluorescence (IF)) and mouse monoclonal antibodies 16L1-312F (diluted 1:200 in IF) and L1-7 (diluted 1:500 in IF) were described previously^23,61,62^. A mouse monoclonal antibody raised against GFP was obtained from Abcam (diluted 1:100 in IF; clone 9F9.F9, cat# ab1218, Cambridge, UK), a mouse monoclonal antibody against CD151 from Bio-Rad (diluted 1:100 in IF; clone 11G5a, cat# MCA1856, Hercules, CA), a mouse monoclonal against integrin α3 from Santa Cruz (diluted 1:100 in IF and 1:1,000 in Western blot (WB); clone A-3, cat# sc-374242, Dallas, TX), a rabbit polyclonal against integrin α6 from Thermo Fisher (diluted 1:200 in IF and 1:1,000 in WB; cat# PA5-12334, Waltham, MA), a mouse monoclonal against L1 from Novus Biologicals (diluted 1:2,000 in WB; CamVir 1, cat# NB100-2732, Centennial, CO) and rabbit monoclonal antibodies against β-Actin (diluted 1:4,000 in WB; clone 13E5, cat# 4970) and CD151 (diluted 1:500 in IF; clone E9M8T, cat# 81626) from Cell Signaling Technology (Danvers, MA).

As secondary antibodies, we employed AlexaFluor488 labelled donkey anti-mouse (cat# A-21202), AlexaFluor594 donkey anti-mouse (cat# A-21203) and AlexaFluor488 goat anti-rabbit (cat# A-11034) from Invitrogen (Carlsbad, CA). Antibody Atto647N goat anti-rabbit (cat# 40839) was obtained from Sigma-Aldrich (St. Louis, MO) and AlexaFluor 594 donkey anti-rabbit (cat# ab150064) from Abcam. STAR RED goat anti-rabbit (cat# STRED-1002) and STAR RED goat anti-mouse (cat# STRED-1001) were obtained from Abberior Instruments (Goettingen, Germany).

GFP-Booster Atto488 (cat# gba488) was obtained from Chromotek (Planegg-Martinsried, Germany).

IRDye secondary antibodies for Western blot detection were 800CW goat anti-mouse (cat# 925-32210), 800CW goat anti-rabbit (cat# 925-32211) and IRDye® 680RD goat anti-rabbit (cat# 925-68071) from Li-Cor (Lincoln, NE).

A plasmid encoding CD151-GFP was described and used previously (pEGFP-C1/CD151^5^).

### Production of pseudoviruses/EdU-PsVs

HPV16 PsVs were prepared as previously described^63^. In brief, expression plasmids carrying codon-optimized L1 and L2^64^ expression vector pShell 16L1/L2wt^65^ were co-transfected with a pcDNA3.1 luciferase reporter plasmid^66^ into HEK 293TT cells using polyethylenimine. For PsVs used in infection assays, the pcDNA3.1 luciferase reporter plasmid was replaced by the promoter-reporter plasmid pGL4.20 containing the HPV16 long control region (LCR) and the HPV16 early promoter regulating the luciferase expression as described earlier^67,68^. For detection of the DNA, EdU-modified PsVs were used. After transfection of pShell 16L1/L2wt and pcDNA3.1 plasmids the cell culture medium was supplemented with 20 μM 5-ethynyl-2′-deoxyuridine (EdU, Click-iT AlexaFluor® 488 Imaging Kit, Thermo Fisher Scientific), to enable the staining of the DNA.

48 hours after transfection, cells were lysed and the pseudoviruses were purified by gradient centrifugation using OptiPrep (Sigma-Aldrich). Quantification of the pseudovirions (viral genome equivalents (vge)) was performed by quantitative PCR using a 7500 Real-Time PCR System and Sequence Detection Software v2.3 (Applied Biosystems, Foster City, CA, USA)^69^.

### Cell culture and transfection

Human immortalized keratinocytes (HaCaT cells) were purchased from Cell Lines Services (Eppelheim, Germany). The human cervical carcinoma cell line (HeLa cells) was obtained from the German Collection of Microorganisms and Cell Cultures ((DSMZ), Braunschweig, Germany). HaCaT cells were maintained in high glucose (4.5 g/l) DMEM (cat# P04-03550 PAN Biotech, Aidenbach, Germany) supplemented with 10% fetal bovine serum (cat# S0615, Biochrom AG, Berlin, Germany) and 1% penicillin-streptomycin working solution (cat# P06-07100, PAN Biotech) at 37 °C and 5% CO_2_. For infection, rescue, and promoter assays, HaCaT and HeLa cells were maintained in DMEM/FCS as above, 1% MEM non-essential amino acid solution (cat# M7145-100 ML, Sigma Aldrich), and 5 µg/ml ciprofloxacin (Fresenius Kabi, Bad Homburg vor der Hoehe, Germany).

Cell line identities were confirmed by Short Tandem Repeat (STR) analysis (Mycrosynth AG, Balgach, Switzerland). Cell lines were tested negative for mycoplasma employing a mycoplasma detection kit (MycoAlert PLUS Mycoplasma Detection Kit, Lonza, Koeln, Germany) and by Microsynth Real-Time PCR analysis (order ID 004264 and 004266; Microsynth, Lindau, Germany).

For transfection with pEGFP-C1/CD151, HaCaT cells were detached by incubation for 10 min in trypsin solution (cat# P10-0231SP, PAN Biotech). Trypsin activity was stopped by adding culture medium. Cells were washed once in DPBS (cat# P04-36500, PAN-Biotech), resuspended in cytomix solution (120 mM KCl, 10 mM KH_2_PO4, 0.15 mM CaCl_2_, 2 mM EGTA, 5 mM MgCl_2_, 25 mM HEPES-KOH, pH 7.6), and 2 × 10^6^ cells were transferred together with 15 µg plasmid DNA into an electroporation cuvette. HaCaTs were electroporated with a Gene pulser Xcell electroporation system (Bio-Rad, Hercules, CA) employing 200 V, 950 μF and 200 Ω. Cells were plated onto poly-L-lysine-coated (PLL) glass-coverslips (~3 ∗ 10^5^ cells/coverslip), incubated for 24 h in culture medium at 37 °C, and used for experiments.

For siRNA knockdown, we employed the following pooled duplex siRNAs: for integrin α6, α6#1 (GGAUAUGCCUCCAGGUUAA[dT][dT]) and α6#2 (CUGUAAGGAUCCGGAAAGA[dT][dT]), and for integrin α3 α3#1 (GCUACAUGAUUCAGCGCAA[dT][dT]) and α3#2 (GUUUGAAGGCUUGGGCAAA[dT][dT]) (Sigma-Aldrich). For control, AllStars Negative control siRNA (Qiagen, Hilden, Germany) was used. For transfection, HaCaT cells were seeded onto PLL-coated glass coverslips or plastic 6-well plates (100,000 cells/well) and incubated overnight in cell culture medium. Then, cells were incubated with 30 nM siRNA using RNAiMAX Lipofectamine transfection reagent (Invitrogen) according to the manufacturer’s instructions. Cells were used 48 h later for experiments.

### L1-cleavage assay

HaCaT cells were seeded onto 6 well-plates (100,000 cells/well), and the next day transfected with siRNAs. Two days later, medium was replaced with medium without antibiotics, supplemented with PsVs (4 ∗ 10^7^ vge per well) and incubated for 24 h at 37 °C. Cells were washed extensively three times with ice-cold PBS (this treatment does not remove plastic-adsorbed viral particles). Afterwards, cells were lysed in Lämmli buffer (63 mM Tris-HCl, 2% w/v SDS, 10% w/v Glycerol, pH 6.8 in ddH_2_O) supplemented with 5% β-mercaptoethanol. Cell lysates were vortexed, boiled for 10 min at 95 °C and stored at −20 °C until SDS-PAGE and Western blot analysis.

### PsV-binding assay

HaCaT cells were seeded onto 6 well-plates (100,000 cells/well), and the next day transfected with siRNAs. Two days later, medium was replaced with ice-cold medium without antibiotics, supplemented without or with PsVs (2 ∗ 10^7^ vge per well) and incubated for 1 h at 4 °C to allow L1 attachment to the cells. Cells were washed extensively three times with ice-cold PBS. Afterwards, cells were scraped from the wells and transferred to a reaction tube, washed three times with ice-cold PBS to remove unbound viral particles and lysed in Lämmli buffer (63 mM Tris-HCl, 2% w/v SDS, 10% w/v Glycerol, pH 6.8 in ddH_2_O) supplemented with 5% β-mercaptoethanol. Cell lysates were vortexed, boiled for 10 min at 95 °C and stored at −20 °C until SDS-PAGE and Western blot analysis.

### SDS-PAGE and western blotting

Samples were analyzed using a 4% stacking gel and a 12% (L1-cleavage assay) or 8% (for integrin detection and PsV-binding assay) polyacrylamide running gel. SDS-PAGE was performed in a MiniPROTEAN Tetra Cell (Bio-Rad) in SDS running buffer (25 mM Tris, 0.1% w/v SDS, 192 mM glycine, pH 8.3 in ddH_2_O). Gel electrophoresis started at 70 V. Voltage was raised to 150 V after samples had left the stacking gel. After the run, gels were incubated for at least 10 min with ice-cold Towbin buffer (25 mM Tris, 192 mM glycine, 20% v/v MeOH, pH 8.3 in ddH_2_O). Nitrocellulose membranes (0.2 µm pore-size, Carl Roth, Karlsruhe, Germany, cat# HP40.1) were also equilibrated in Towbin buffer for 30 min at 4 °C. Then, proteins were blotted in a MiniPROTEAN Tetra Cell equipped with Mini Trans-Blot Module in cooled Towbin buffer under constant agitation. Transfer was performed for 45 min (L1-cleavage assay and PsV-binding assay) or for 2 h (for integrin detection) at 100 V. Afterwards, membranes were washed in PBS and blocked for 1 h in 50% Odyssey Blocking Buffer (Li-Cor, cat# 927–40000) in PBS. Then, membranes were incubated with primary antibodies diluted in 50% Odyssey Blocking Buffer in PBS supplemented with 0.1% Tween-20 for 1 h at RT or overnight at 4 °C under constant agitation. Membranes were washed four times with PBS-T (0.1% Tween-20 in PBS) and incubated with secondary antibodies diluted 1:10,000 in 50% Odyssey Blocking Buffer in PBS supplemented with 0.1% Tween-20 for 1 h at RT. Afterwards, membranes were washed three times in PBS-T and one time in PBS. Blots were imaged with a Li-Cor Odyssey Classic Imaging System using the 700 nm and 800 nm channels. Detected bands were quantified using ImageJ software and corrected for local background.

### Infection and promoter assay

For luciferase-based infection and promoter assays, HaCaT cells were seeded in 24-well plates (30,000 and 70,000 for the infection and promoter assay per well, respectively) and cultured in medium without antibiotics. The next day cells were transfected with siRNA. For infection assays, 48 hours after siRNA transfection the cells were infected with approximately 1 ∗ 10^7^ vge per well and incubated for additional 24 hours prior to analysis. For promoter assays, 24 hours after siRNA transfection, cells were transfected with pGL4.20 HPV16 LCR luciferase reporter plasmid^67,68^ using polyethylenimine. After 24 hours, luciferase activity was measured.

For the analysis of luciferase activity, cells were washed once with phosphate-buffered saline (PBS) and lysed in Cell Culture Lysis Reagent (Promega, Fitchburg, MA, USA). After 30 minutes of shaking in 250 μl lysis buffer, the cells were transferred to tubes, centrifuged, and 150 μl of the supernatant was measured for luciferase counts. For normalization of the activity to the number of cells, we determined the activity of the constitutively expressed lactate dehydrogenase (LDH) using the CytoTox-ONE™ Homogeneous Membrane Integrity Assay (Promega). Both, luciferase and LDH activities were measured by the Tristar LB 941 luminometer (Berthold Technologies, Bad Wildbad, Germany).

### CD151 rescue

HeLa cells were transfected with CD151 specific siRNA (CACAUACAGGUGCUCAAUAAAdTdT (Sigma Aldrich)) targeting the 3′-untranslated region (3′UTR) of the CD151 gene but not affecting expression of the protein from the transfected CD151-encoding plasmids^5^ using RNAiMAX Lipofectamine transfection reagent (Invitrogen) according to the manufacturer’s instructions. After 24 hours, cells were transfected with plasmids encoding for CD151 WT^26^, pEGFP-C1/CD151 or control plasmid pcDNA3.1 (Invitrogen, San Diego, CA) using Lipofectamine 2000 (Invitrogen). After 24 h, the cells were incubated with 100 vge per cell for another day and the infection rate was measured.

### Immunostaining

Untransfected, pEGFP-C1/CD151 or siRNA transfected HaCaT cells were plated onto PLL-coated glass-coverslips that were placed into 6-well plates. After one, or in the case of siRNA transfection two days, cells were incubated for 3–5 h without or with 4 ∗ 10^7^ vge per well in culture medium without antibiotics. Afterwards, cells were extensively washed twice in PBS. Cells were immunostained directly or after membrane sheet generation. Membrane sheets were generated in ice-cold sonication buffer (120 mM potassium glutamate, 20 mM potassium acetate, 10 mM EGTA, 20 mM HEPES, pH 7.2) essentially as previously described^70^, with the difference that several 100 ms ultrasound pulses were applied at different coverslip locations.

For immunostaing, cells or membrane sheets were fixed in 4% PFA in PBS for 30 min at room temperature. The fixation solution was removed and residual PFA was quenched with 50 mM NH_4_Cl in PBS for 30 min. Then, cells and membrane sheets were permeabilized with 0.2% Triton X-100 in PBS for 2 min and 1 min, respectively. In experiments in which EdU-PsVs were used one additional step was performed, which was click-labeling of the plasmid DNA with fluorescein for 30 min at RT according to the manufacturer’s instructions (EdU Click 488 kit, Carl Roth, cat# 7773.1). Afterwards, the sample was blocked with 3% BSA in PBS for 30 min.

In experiments in which the rabbit monoclonal anti-CD151 antibody was used in combination with the monoclonal antibody against integrin α3, methanol fixation was employed. Samples were fixed in 100% methanol for 15 min at −20 °C, washed three times with PBS and blocked.

Staining with primary antibodies was performed in 3% BSA for 1 h at room temperature (RT) or in knockdown experiments and for triple co-localization overnight at 4 °C. Afterwards, samples were washed in PBS and incubated with secondary antibodies (diluted 1:200 in 3% BSA in PBS) for 1 h at RT. For F-Actin staining, Phalloidin-iFluor647 (Abcam, cat# ab176759) was used. According to the manufacturer’s instructions, a 100× stock solution in DMSO was prepared that was added at a 1:100 dilution to the solution with secondary antibodies. Finally, samples were washed in PBS and mounted onto microscopy slides with ProLong® Gold antifade mounting medium (Invitrogen, cat# P36930). Samples were cured for 24 h and sealed with nail varnish.

### STED super-resolution and confocal microscopy

For STED and confocal microscopy, a 4-channel easy3D super-resolution STED optics module (Abberior Instruments) coupled with an Olympus IX83 confocal microscope (Olympus, Tokyo, Japan) and equipped to an UPlanSApo 100 × (1.4 NA) objective (Olympus, Tokyo, Japan) was used. Atto488 and Alexa488 were excited with a 485 nm laser and recorded with combined 500–520 nm and 532–558 nm filters. Alexa594 was excited with a 561 nm laser and recorded with a 580–630 nm filter. Atto647N, STAR RED and iFluor647 were excited with a 640 nm laser and detected with a 650–720 nm filter. The pinhole size was set to 60 µm. For STED microscopy, pulsed STED lasers 595 nm (for Alexa488 and Atto488) and 775 nm (for Alexa594, Atto647N, and STAR RED) were used for depletion. STED images were recorded via a time-gated detection with 0.75 ns delay and 8 ns gate width. Depending on the experiment, pixel size was set to 20–40 nm.

For intracellular vesicles visualized with the L1–7-antibody (Fig. 1B), the cell body was recorded in the focal plane where most vesicles were visible. In all other experiments, the focal plane was adjusted to the basal membrane area.

### Epifluorescence microscopy

After immunostaining, samples were washed with PBS and imaged directly in PBS containing 1-(4-tri-methyl-ammonium-phenyl)−6-phenyl-1,3,5-hexatriene p-toluene-sulfonate (TMA-DPH; cat# T-204, Invitrogen) for the visualization of membranes. Imaging was performed with an Olympus IX81 fluorescence microscope essentially as described previously^32^.

### Image analysis

For image analysis the program ImageJ was used. Regions of interest (ROIs) were placed in one reference channel and propagated to the other channel(s). For fluorescence intensity measurements, mean intensity values within the ROIs were corrected for local background intensity from a ROI placed outside of the cell or membrane sheet.

For analyzing maxima or vesicle parameters (density, size, distances and fluorescence intensity), a custom ImageJ macro was used. The macro is based on the ImageJ function “Find Maxima” which was employed for the detection of maxima. Prior to analysis, images were smoothed with a Gaussian blur (σ = 1) to reduce pixel noise and thereby improve maxima detection. The coordinates of detected maxima were used for further analysis. For maxima intensity, a 2 pixel radius circular ROI was placed at the maxima location and the mean intensity value was measured. Maxima with a low mean intensity (depending on the experiment <1–3 a.u.) were excluded from the further analysis. For inter-maxima distance analysis, the nearest neighbor of each maxima in the same or the other channel was calculated. To this end, first all distances between one and the other maxima were calculated, followed by extracting the shortest one. The maxima size was determined by applying a vertical and a horizontal 31 × 3 pixel line scan at the maxima position. A Gaussian function was fitted to the intensity distribution. The full width at half maximum (FWHM) of the Gaussian was taken as maxima size. Depending on the best fit quality, the size value was taken either from the horizontal or vertical scan. Maxima with a fit quality of R^2^ < 0.8 and a non-centered peak (not in the middle third of the linescan) were excluded. For each individual cell or membrane sheet, all maxima parameter values were averaged. The maxima density was obtained by relating the number of maxima to the size of the analyzed area.

For the characterization of the platform area, we placed a 1 µm × 1 µm ROI centered to the PsV location. We counted only the brighter maxima, setting 8, 11, 15, and 14 intensity counts as threshold for CD151-rabbit antibody-, CD151-mouse antibody-, integrin α3-antibody- and integrin α6-antibody stained maxima, respectively.

Colocalization was also analyzed with a custom ImageJ macro. The macro is based on ImageJs built-in autothresholding function. A value of three intensity counts was substracted to avoid selecting background in images with weak signal. Images were smoothed with a Gaussian blur (σ = 1) to enhance edge detection. Within the ROI, the image was segmented with the autothresholding method “Mean”. The segmented area from one channel was compared to the segmented area from a second (for double colocalization) and a second/third channel (for triple colocalization), and the overlapping area was calculated. Colocalization was calculated as the ratio of overlapping pixels to total pixels. Alternatively, a custom ImageJ macro was used for calculation of the Pearson correlation coefficient (PCC) from ROIs placed onto cells or membrane sheets.

## Supplementary information

Supplementary Information.

## References

[1] Charrin S, Jouannet S, Boucheix C, Rubinstein E (2014). Tetraspanins at a glance. Journal of cell science.

[2] Monk PN, Partridge LJ (2012). Tetraspanins: gateways for infection. Infectious disorders drug targets.

[3] Fast LA, Lieber D, Lang T, Florin L (2017). Tetraspanins in infections by human cytomegalo- and papillomaviruses. Biochemical Society transactions.

[4] Spoden G (2008). Clathrin- and caveolin-independent entry of human papillomavirus type 16–involvement of tetraspanin-enriched microdomains (TEMs). PloS one.

[5] Scheffer KD (2013). Tetraspanin CD151 Mediates Papillomavirus Type 16 Endocytosis. Journal of Virology.

[6] Florin L, Lang T (2018). Tetraspanin Assemblies in Virus Infection. Frontiers in immunology.

[7] Earnest JT, Hantak MP, Park J-E, Gallagher T (2015). Coronavirus and influenza virus proteolytic priming takes place in tetraspanin-enriched membrane microdomains. Journal of Virology.

[8] Zona L (2013). HRas signal transduction promotes hepatitis C virus cell entry by triggering assembly of the host tetraspanin receptor complex. Cell host & microbe.

[9] Bruening J (2018). Hepatitis C virus enters liver cells using the CD81 receptor complex proteins calpain-5 and CBLB. PLoS Pathogens.

[10] Stipp CS (2010). Laminin-binding integrins and their tetraspanin partners as potential antimetastatic targets. Expert reviews in molecular medicine.

[11] Evander M (1997). Identification of the alpha6 integrin as a candidate receptor for papillomaviruses. Journal of Virology.

[12] McMillan NA, Payne E, Frazer IH, Evander M (1999). Expression of the alpha6 integrin confers papillomavirus binding upon receptor-negative B-cells. Virology.

[13] Yoon CS, Kim KD, Park SN, Cheong S (2001). W. alpha(6) Integrin is the main receptor of human papillomavirus type 16 VLP. Biochemical and biophysical research communications.

[14] Aksoy P, Abban CY, Kiyashka E, Qiang W, Meneses PI (2013). HPV16 infection of HaCaTs is dependent on β4 integrin, and α6 integrin processing. Virology.

[15] Giroglou T, Florin L, Schäfer F, Streeck RE, Sapp M (2001). Human Papillomavirus Infection Requires Cell Surface Heparan Sulfate. Journal of Virology.

[16] Shafti-Keramat S (2003). Different heparan sulfate proteoglycans serve as cellular receptors for human papillomaviruses. Journal of Virology.

[17] Huang H-S, Lambert PF (2012). Use of an *in vivo* animal model for assessing the role of integrin α(6)β(4) and syndecan-1 in early steps in papillomavirus infection. Virology.

[18] Surviladze Z, Sterkand RT, Ozbun MA (2015). Interaction of human papillomavirus type 16 particles with heparan sulfate and syndecan-1 molecules in the keratinocyte extracellular matrix plays an active role in infection. The Journal of general virology.

[19] Harburger DS, Calderwood DA (2009). Integrin signalling at a glance. Journal of cell science.

[20] Nemerow GR, Cheresh DA (2002). Herpesvirus hijacks an integrin. Nature cell biology.

[21] Abban CY, Meneses PI (2010). Usage of heparan sulfate, integrins, and FAK in HPV16 infection. Virology.

[22] Cerqueira C, Samperio Ventayol P, Vogeley C, Schelhaas M (2015). Kallikrein-8 Proteolytically Processes Human Papillomaviruses in the Extracellular Space To Facilitate Entry into Host Cells. Journal of Virology.

[23] Sapp M (1994). Analysis of type-restricted and cross-reactive epitopes on virus-like particles of human papillomavirus type 33 and in infected tissues using monoclonal antibodies to the major capsid protein. The Journal of general virology.

[24] Yauch RL, Berditchevski F, Harler MB, Reichner J, Hemler ME (1998). Highly Stoichiometric, Stable, and Specific Association of Integrin α3β1 with CD151 Provides a Major Link to Phosphatidylinositol 4-Kinase, and May Regulate Cell Migration. Molecular Biology of the Cell.

[25] Yauch RL, Kazarov AR, Desai B, Lee RT, Hemler ME (2000). Direct Extracellular Contact between Integrin α 3 β 1 and TM4SF Protein CD151. The Journal of biological chemistry.

[26] Zhang XA (2002). Function of the Tetraspanin CD151–α6β1 Integrin Complex during Cellular Morphogenesis. Molecular Biology of the Cell.

[27] Uhlén M (2015). Proteomics. Tissue-based map of the human proteome. Science (New York, N.Y.).

[28] Mikuličić, S. et al. ADAM17-dependent signaling is required for oncogenic human papillomavirus entry platform assembly. *eLife* **8**; 10.7554/eLife.44345 (2019). 10.7554/eLife.44345 PMC6557631 31107240

[29] Willig KI, Rizzoli SO, Westphal V, Jahn R, Hell SW (2006). STED microscopy reveals that synaptotagmin remains clustered after synaptic vesicle exocytosis. Nature.

[30] Merklinger, E. *et al*. The packing density of a supramolecular membrane protein cluster is controlled by cytoplasmic interactions. *eLife* **6**; 10.7554/eLife.20705 (2017). 10.7554/eLife.20705 PMC5536946 28722652

[31] Zuidscherwoude M (2015). The tetraspanin web revisited by super-resolution microscopy. Scientific reports.

[32] Homsi Y (2014). The extracellular δ-domain is essential for the formation of CD81 tetraspanin webs. Biophysical journal.

[33] Selinka H-C, Giroglou T, Nowak T, Christensen ND, Sapp M (2003). Further evidence that papillomavirus capsids exist in two distinct conformations. Journal of Virology.

[34] Becker, M., Greune, L., Schmidt, M. A. & Schelhaas, M. Extracellular Conformational Changes in the Capsid of Human Papillomaviruses Contribute to Asynchronous Uptake into Host Cells. *Journal of Virology* **92**; 10.1128/JVI.02106-17 (2018). 10.1128/JVI.02106-17 PMC5952151 29593032

[35] Schelhaas, M. *et al*. Entry of Human Papillomavirus Type 16 by Actin-Dependent, Clathrin- and Lipid Raft-Independent Endocytosis. *Plos Pathogens* **8**; 10.1371/journal.ppat.1002657 (2012). 10.1371/journal.ppat.1002657 PMC3334892 22536154

[36] Ozbun MA (2019). Extracellular events impacting human papillomavirus infections: Epithelial wounding to cell signaling involved in virus entry. Papillomavirus research (Amsterdam, Netherlands).

[37] Mikuličić S, Florin L (2019). The endocytic trafficking pathway of oncogenic papillomaviruses. Papillomavirus research (Amsterdam, Netherlands).

[38] Lang T, Rizzoli SO (2010). Membrane protein clusters at nanoscale resolution: more than pretty pictures. Physiology (Bethesda, Md.).

[39] Raff AB (2013). The evolving field of human papillomavirus receptor research: a review of binding and entry. Journal of Virology.

[40] Surviladze Z, Dziduszko A, Ozbun MA (2012). Essential roles for soluble virion-associated heparan sulfonated proteoglycans and growth factors in human papillomavirus infections. PLoS Pathogens.

[41] Ivaska J (2012). Unanchoring integrins in focal adhesions. Nature cell biology.

[42] Berditchevski F (2001). Complexes of tetraspanins with integrins: more than meets the eye. Journal of cell science.

[43] Winterwood NE, Varzavand A, Meland MN, Ashman LK, Stipp CS (2006). A critical role for tetraspanin CD151 in alpha3beta1 and alpha6beta4 integrin-dependent tumor cell functions on laminin-5. Molecular Biology of the Cell.

[44] Takeda Y (2007). Deletion of tetraspanin Cd151 results in decreased pathologic angiogenesis *in vivo* and *in vitro*. Blood.

[45] Yang XH (2008). CD151 accelerates breast cancer by regulating alpha 6 integrin function, signaling, and molecular organization. Cancer research.

[46] Berditchevski F (2001). Analysis of the CD151-alpha3beta1 integrin and CD151-tetraspanin interactions by mutagenesis. The Journal of biological chemistry.

[47] Kazarov AR, Yang X, Stipp CS, Sehgal B, Hemler ME (2002). An extracellular site on tetraspanin CD151 determines α3 and α6 integrin–dependent cellular morphology. J Cell Biol.

[48] Selinka H-C (2007). Inhibition of transfer to secondary receptors by heparan sulfate-binding drug or antibody induces noninfectious uptake of human papillomavirus. Journal of Virology.

[49] Bienkowska-Haba M, Patel HD, Sapp M (2009). Target cell cyclophilins facilitate human papillomavirus type 16 infection. PLoS Pathogens.

[50] Richards KF, Bienkowska-Haba M, Dasgupta J, Chen XS, Sapp M (2013). Multiple Heparan Sulfate Binding Site Engagements Are Required for the Infectious Entry of Human Papillomavirus Type 16. Journal of Virology.

[51] Culp TD, Budgeon LR, Marinkovich MP, Meneguzzi G, Christensen ND (2006). Keratinocyte-secreted laminin 5 can function as a transient receptor for human papillomaviruses by binding virions and transferring them to adjacent cells. Journal of Virology.

[52] Payne E, Bowles MR, Don A, Hancock JF, McMillan NA (2001). Human papillomavirus type 6b virus-like particles are able to activate the Ras-MAP kinase pathway and induce cell proliferation. Journal of Virology.

[53] Fothergill T, McMillan NAJ (2006). Papillomavirus virus-like particles activate the PI3-kinase pathway via alpha-6 beta-4 integrin upon binding. Virology.

[54] Li E, Stupack D, Klemke R, Cheresh DA, Nemerow GR (1998). Adenovirus endocytosis via alpha(v) integrins requires phosphoinositide-3-OH kinase. Journal of Virology.

[55] Hodivala-Dilke KM, Michael DiPersio C, Kreidberg JA, Hynes RO (1998). Novel Roles for α3β1 Integrin as a Regulator of Cytoskeletal Assembly and as a Trans-dominant Inhibitor of Integrin Receptor Function in Mouse Keratinocytes. J. Cell. Biol..

[56] Wang Z (1999). (Alpha)3(beta)1 integrin regulates epithelial cytoskeletal organization. Journal of cell science.

[57] Shigeta M (2003). CD151 regulates epithelial cell–cell adhesion through PKC- and Cdc42-dependent actin cytoskeletal reorganization. J. Cell. Biol..

[58] Lee J, Lee J, Choi C, Kim JH (2019). Blockade of integrin α3 attenuates human pancreatic cancer via inhibition of EGFR signalling. Scientific reports.

[59] Spoden G (2013). Human Papillomavirus Types 16, 18, and 31 Share Similar Endocytic Requirements for Entry. Journal of Virology.

[60] Hantak, M. P., Qing, E., Earnest, J. T. & Gallagher, T. Tetraspanins: Architects of Viral Entry and Exit Platforms. *Journal of Virology* 93; 10.1128/JVI.01429-17 (2019). 10.1128/JVI.01429-17 PMC6401424 30567993

[61] Bergsdorf C, Beyer C, Umansky V, Werr M, Sapp M (2003). Highly efficient transport of carboxyfluorescein diacetate succinimidyl ester into COS7 cells using human papillomavirus-like particles. FEBS Letters.

[62] Knappe M (2007). Surface-exposed amino acid residues of HPV16 L1 protein mediating interaction with cell surface heparan sulfate. The Journal of biological chemistry.

[63] Buck CB, Pastrana DV, Lowy DR, Schiller JT (2004). Efficient intracellular assembly of papillomaviral vectors. Journal of Virology.

[64] Leder C, Kleinschmidt JA, Wiethe C, Müller M (2001). Enhancement of Capsid Gene Expression: Preparing the Human Papillomavirus Type 16 Major Structural Gene L1 for DNA Vaccination Purposes. Journal of Virology.

[65] Buck CB (2006). Carrageenan is a potent inhibitor of papillomavirus infection. PLoS Pathogens.

[66] Schneider MA, Spoden GA, Florin L, Lambert C (2011). Identification of the dynein light chains required for human papillomavirus infection. Cellular microbiology.

[67] Schneider MA (2013). The transcription factors TBX2 and TBX3 interact with human papillomavirus 16 (HPV16) L2 and repress the long control region of HPVs. Journal of Virology.

[68] Wüstenhagen E (2018). The Myb-related protein MYPOP is a novel intrinsic host restriction factor of oncogenic human papillomaviruses. Oncogene.

[69] Spoden GA (2012). Polyethylenimine is a strong inhibitor of human papillomavirus and cytomegalovirus infection. Antimicrobial agents and chemotherapy.

[70] Zilly FE (2011). Ca2+ induces clustering of membrane proteins in the plasma membrane via electrostatic interactions. The EMBO journal.

