# Supplementary material for: Anatomy of a viral entry platform differentially functionalized by integrins α3 and α6
Source: Sci Rep. 2020 Mar 24;10:5356. doi: 10.1038/s41598-020-62202-9 (PMC7093462; doi:10.1038/s41598-020-62202-9)
Supplement: Supplementary file 1 — Supplementary Information. [file 41598_2020_62202_MOESM1_ESM.pdf]

Supplementary Information

Anatomy of a viral entry platform differentially functionalized by integrins α3 and α6

Jérôme Finke[1], Snježana Mikuličić[2], Anna-Lena Loster[2], Alexander Gawlitza[2], Luise Florin[2], Thorsten Lang[1*]

[1]Department of Membrane Biochemistry, Life & Medical Sciences Institute (LIMES), University of Bonn, Carl-Troll-Straße 31, 53115 Bonn, Germany, [2]Institute for Virology and Research Center for Immunotherapy (FZI), University Medical Center of the Johannes Gutenberg University Mainz, Obere Zahlbacher Straße 67, 55131 Mainz, Germany

[*]Correspondence should be addressed to thorsten.lang@uni-bonn.de

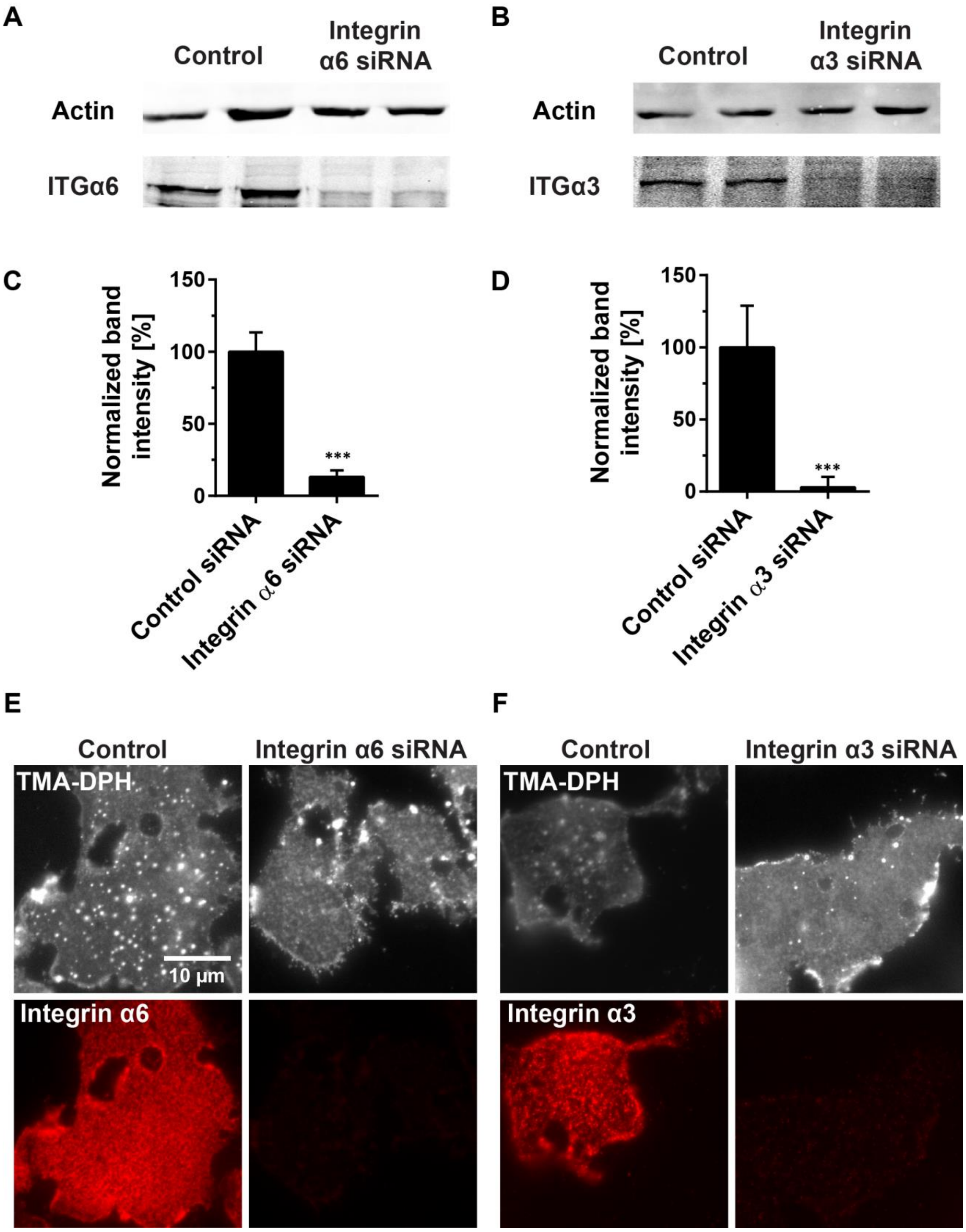

Figure S1. *Integrin knockdown efficiency.*
(A - D) HaCaT cells were transfected with control, integrin α6 or integrin α3 siRNA. After 2 days, cell lysates were analyzed for (A and C) integrin α6 or (B and D) integrin α3 by Western blot analysis using actin as loading control. Two lysates per condition are shown. For full Western blots see Figures S18 – S21. Integrin band intensities were related to actin band intensities. The average of the control lysates for each biological replicate was set to 100%. Values are given as means ± SD (n = 6 - 8 lysates collected from 3 - 4 biological replicates). The unpaired Student's t-test compares the control to the knockdown (***, $p < 0.001$). (E and F). Example images from an experiment in which membrane sheets from control and knockdown cells were generated and immunostained for (E) Integrin α6 or (F) integrin α3. Upper panels show membranes visualized by the membrane dye TMA-DPH, lower panels the respective integrin immunostainings. Images are displayed employing greyscale (TMA-DPH) and red (integrins) linear lookup tables. For E and F different scalings were applied, although all panels from one channel in E or F have the same arbitrary scaling. Please note that after knockdown the integrin antibodies hardly recognize any protein.

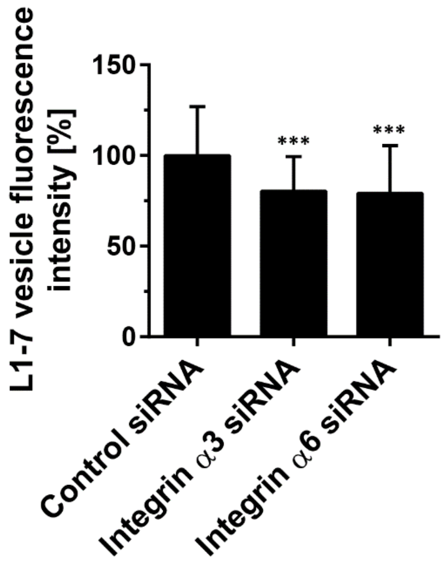

Figure S2. *L1-7 vesicle staining intensity.*
Fluorescence intensity of L1-7 positive vesicles as counted in Figure 1C. Individual vesicle intensity values were averaged per cell. Cells without vesicles were excluded from the analysis. Values are given as means ± SD (n = 58 - 60 cells collected from three biological replicates). Unpaired Student’s t-test, comparing control to knockdown conditions (***, $p < 0.001$).

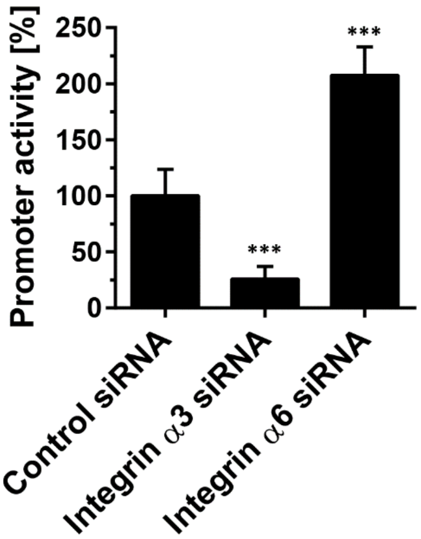

Figure S3. *Direct plasmid transfection reveals secondary integrin knockdown effects on promoter activity.*
The encapsidated plasmid used for Figure 1D was directly transfected into HaCaT cells. This circumvents the viral entry pathway and allows for analyzing secondary integrin knockdown effects on the viral promoter activity that are not related to the viral infection pathway. In brief, we transfected the siRNAs into HaCaT cells, and 1 day later the plasmid. After another day, we measured luciferase and lactate dehydrogenase activity. For normalization to cell number luciferase activity was normalized to dehydrogenase activity. Values are given as means ± SD (n = 12 technical replicates collected from 3 biological replicates). The knockdown conditions show that in the absence of integrin α3, luciferase activity is strongly diminished, whereas in the absence of integrin α6 its activity is strongly increased. Normalization of infection rates from Figure 1D to these secondary effects yield infection rates of 48% and 16% after integrin α3 and integrin α6 knockdown, respectively. The unpaired Student's t-test compares the control to the knockdown conditions (***, $p < 0.001$).

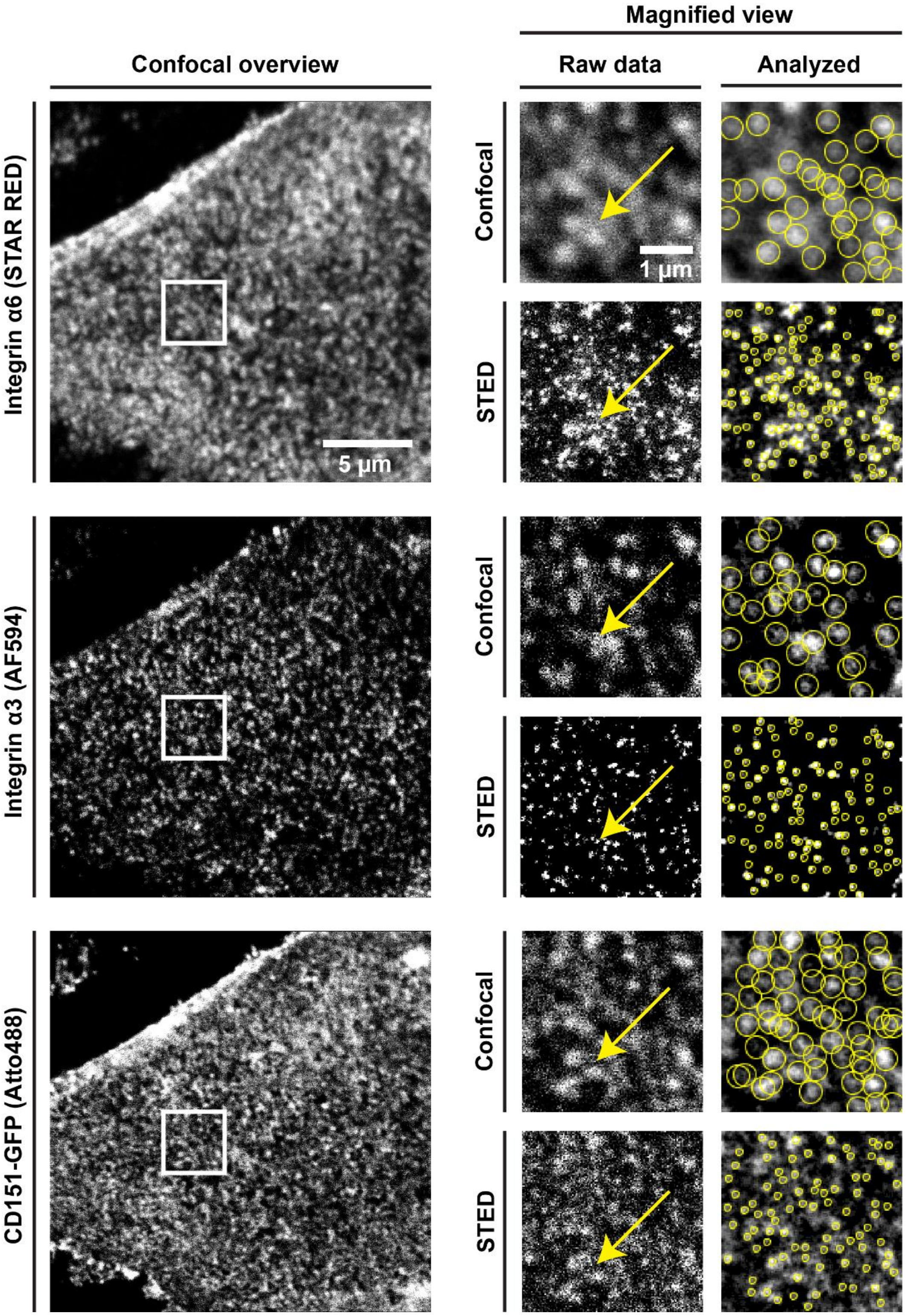

Figure S4. *Illustration of the gain in resolution by STED microscopy.*
Membrane sheets from cells overexpressing CD151-GFP were fixed and stained for integrin α3 and integrin α6. CD151-GFP fluorescence was enhanced by nanobodies. From the confocal channels, overviews and magnified views are shown, and from the magnified views also the STED micrographs. Magnified views in the left column show raw data, the right column illustrates smoothed images that were used for maxima detection. Circles indicate locations where maxima were detected. Images are shown using a linear greyscale lookup table employing an arbitrary scaling. Please note that STED microscopy allows for resolving larger spots into smaller entities (arrows point to a patch that is better resolved in the integrin channels).

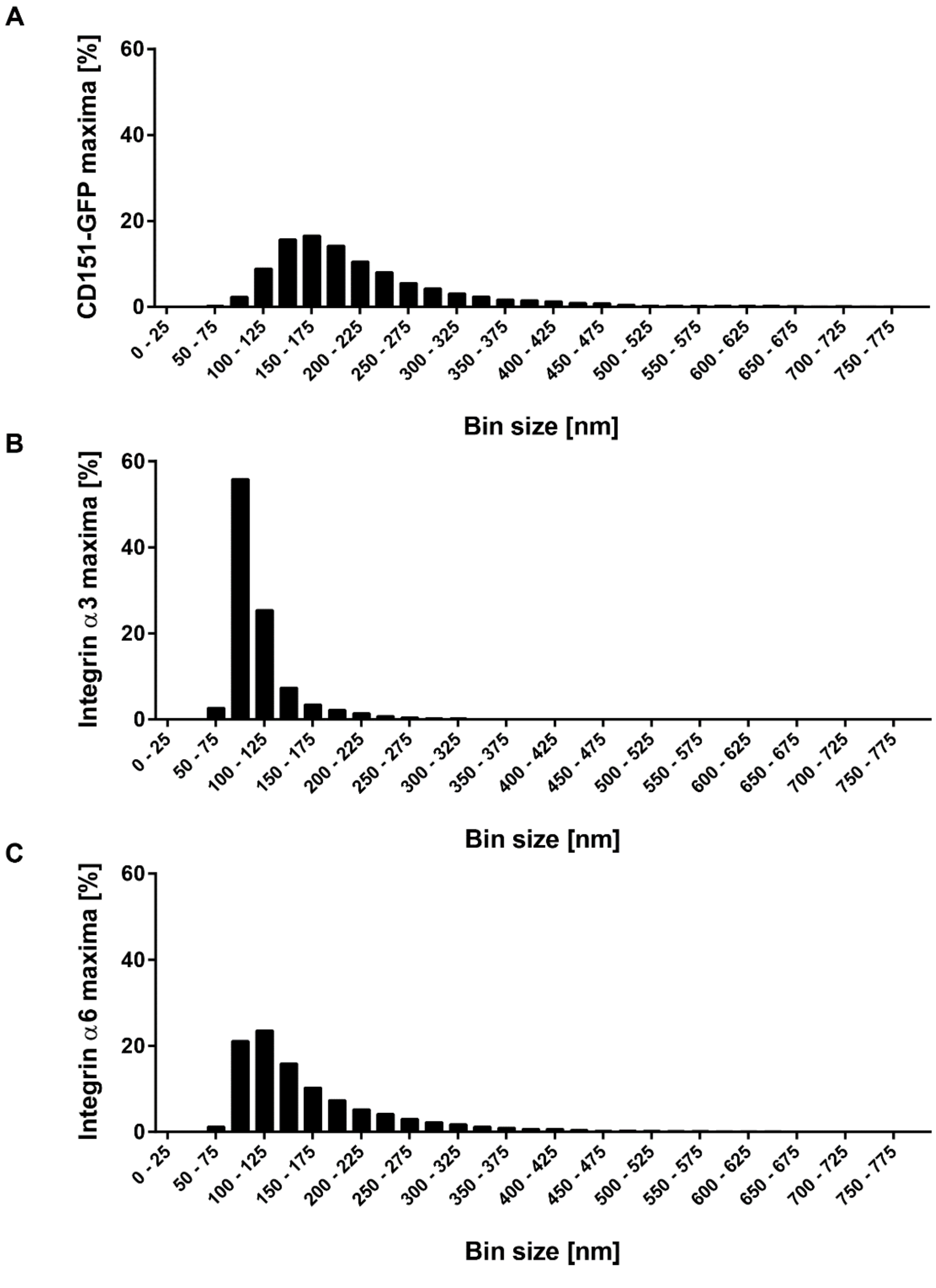

Figure S5. *Histogram of sizes of CD151, integrin α3 and integrin α6 maxima.*

From Figure 3B, we show for the individual channels the distribution of maxima sizes as determined by Gaussian fitting. A Gaussian function was fitted to the intensity profile obtained from a 31-pixel long linescan, from which the central pixel was positioned to the maxima detected by the algorithm. Only maxima were included if the R^2 value of the fit was > 0.8, and if the determined center position of the Gaussian peak was within the central third of the linescan. As a result, the analysis includes 62.2% (22623 maxima), 39.1% (20209 maxima) and 42.7% (8300 maxima) of the initially detected integrin α3, integrin α6 and CD151 maxima, respectively.

The histograms show lognormal distributions, in particular for the smaller integrin α3 maxima. Moreover, no sizes smaller than 50 nm are detected. This points towards the possibility that our STED microscope cannot resolve size differences within the fraction of smaller maxima and that the resolution limit does not reach the range of 50 nm. For verification, we determined the point spread function (PSF) of our STED system (Figure S6).

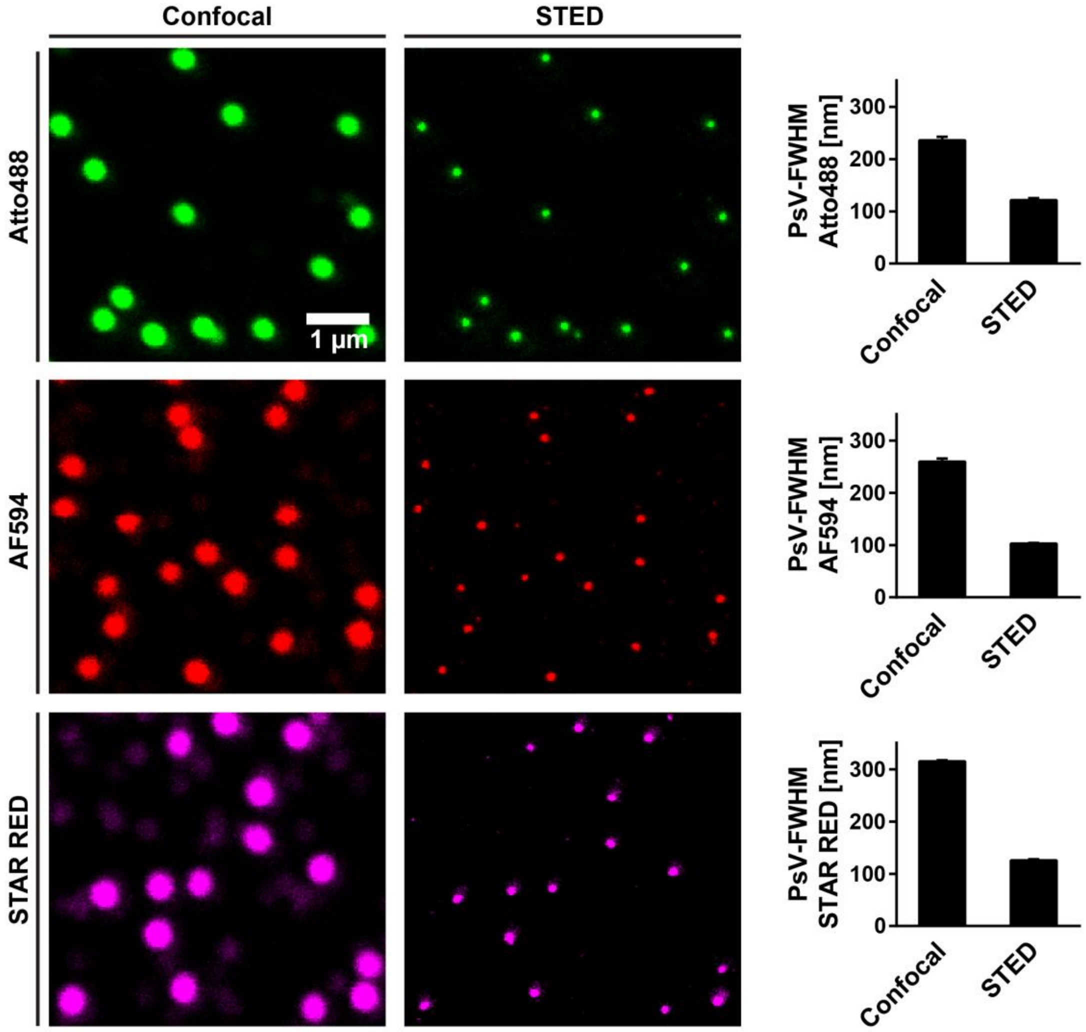

Figure S6. *Determination of the point spread functions of the STED system.*
For determination of the point spread function we used PsVs for calibration. Papillomaviruses have a physical size of ≈ 60 nm [1]. After staining with primary and secondary antibodies, we estimate that the 'shell of fluorophores' increases the diameter by about 20 nm (as has been observed before for microtubuli [2]) to roughly 80 nm. For sample preparation, one µl of PsVs, corresponding to ~ $2*10^7$ viral genome equivalents (vge), was diluted in 1 ml of DMEM and the solution was pipetted onto a poly-L-lysine coated glass-coverslip. PsVs were allowed to adsorb overnight at 4°C and stained as in Figure 6, just omitting the permeabilisation step and CD151 antibodies, and using instead of the K75 the 16L1-312F antibody. Secondary antibodies were labelled by the dyes Atto488 (used in Figure 3 for CD151-GFP labelling; linear green lookup table), AF594 (used in Figure 3 for integrin α3 labelling; linear red lookup table) and STAR RED (used in Figure 3 for integrin α6 labelling; linear magenta lookup table). PsVs were imaged employing the same STED laser powers and excitation laser powers as for the maxima analysis in Figure 3. All Images are displayed at arbitrary intensity scalings. Maxima were analysed as in Figure 3. Values are given as means ± SD (n = 10 images per dye, each obtained from one coverslip; in total, the analysis includes 582 - 1461 maxima per dye). PSFs were calculated using the formula PSF = sqrt(measured size^2 – physical size^2), with a value of 80 nm taken as the real physical size. In the STED mode, the sizes of the PsVs are reduced from 236 - 315 nm to 103 – 127 nm. The calculated PSFs are 91 nm, 64 nm, and 97 nm for the CD151-GFP, integrin α3, and integrin α6 channels, respectively. Deconvolution with these PSFs yields physical sizes of the maxima of 189 nm (CD151), 84 nm (integrin α3), and 123 nm (integrin α6).

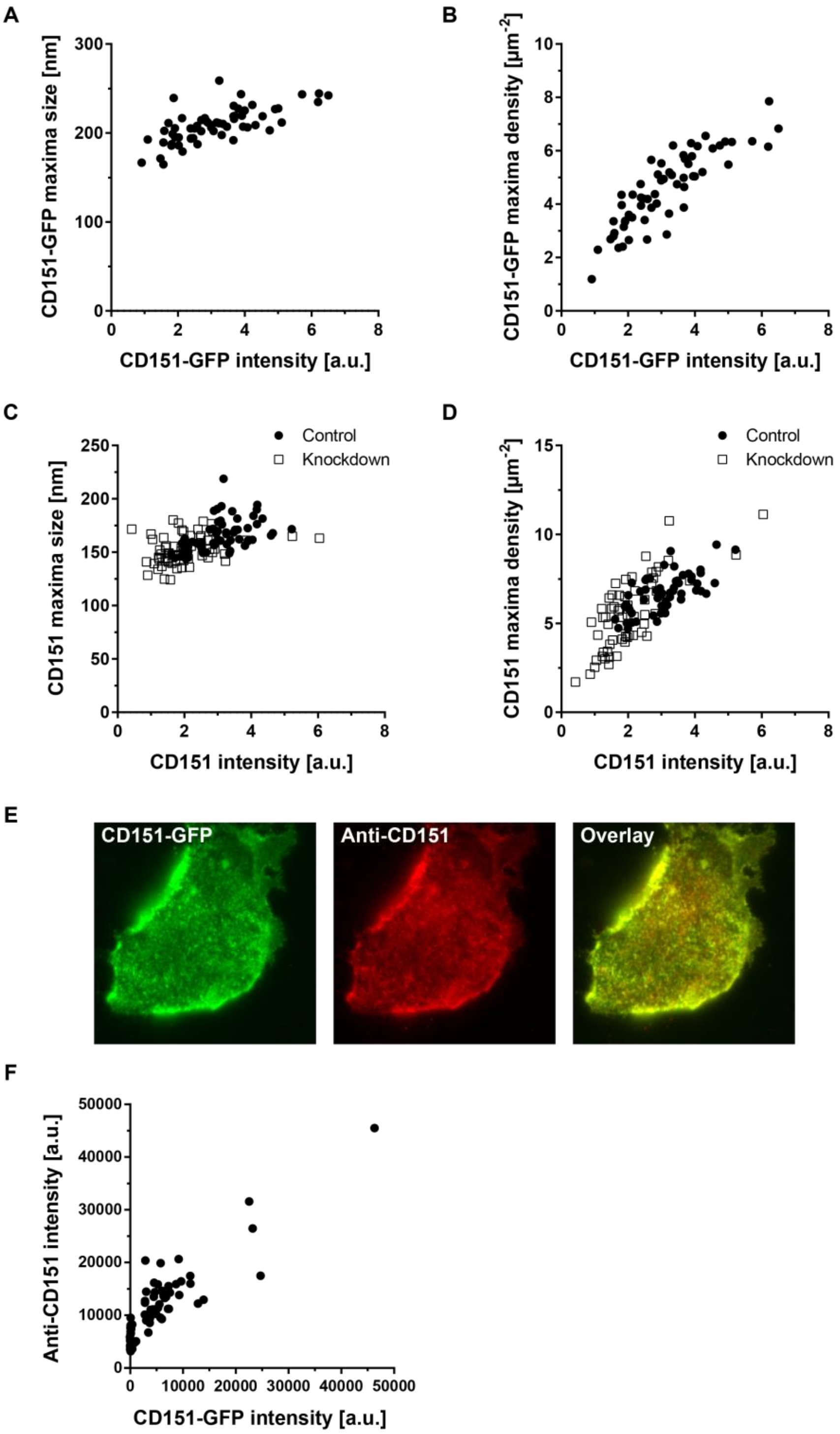

Figure S7. *No difference in clustering behaviour between overexpressed and endogenous CD151.*
(A-D) Dependence of maxima size and maxima density from the expression level, comparing endogenous and overexpressed CD151. (A and B) Maxima size (A) and density (B) from the overexpression experiment shown in Figure 3. Only CD151-GFP was visualized and not endogenous CD151. (C and D) Maxima size (C) and density (D) from the knockdown experiment shown in Figure 6A-C. After integrin α6 knockdown, the level of endogenous CD151 decreases by 33 % (Figure 6B). In the different conditions (overexpressed, low and normal endogenous level), doubling of the expression level roughly increases the maxima size by 10 – 15 % and the density by 30 – 50 %. Please note that absolute maxima size and density values cannot be compared throughout experiments as different staining and imaging conditions are applied.
(E) CD151-GFP was transfected into HaCaT cells. After one day, membrane sheets were generated, fixed, permeabilized, immunostained for CD151 and imaged by epi-fluorescence microscopy. In case that overexpressed CD151-GFP and endogenous CD151 would segregate into different domains, the

endogenous CD151 domains would not overlap with GFP. Images are displayed with linear green (CD151-GFP) and red (anti-CD151) lookup tables at arbitrary intensity scalings. The visual impression and the high Pearson correlation coefficient between the two channels (0.75 ± 0.09; value is given as mean ± SD; n = 36 membrane sheets collected from three biological replicates) suggest that both CD151-GFP and endogenous CD151 locate to the same domains. In fact, the Pearson correlation coefficient is higher than the value from an earlier experiment in which a double tagged protein was used (0.63; [3]), which can be taken as a reference for perfect co-localization.

(F) Plotting of CD151 immunostaining intensity versus GFP fluorescence intensity. In addition to the 36 membrane sheets analysed in E, supplementary membrane sheets, also without CD151-GFP, were included. The average immunostaining intensity of these membrane sheets marks the endogenous CD151. The graph suggests that upon overexpression the total CD151 expression level (endogenous + overexpressed) increases on average by 2.5 fold when compared to the endogenous level. In this experiment, compared to Figure 3, it was necessary to image in the GFP-channel brighter membrane sheets, in order to have comparable amounts of endogenous and overexpressed CD151 in the cell membrane.

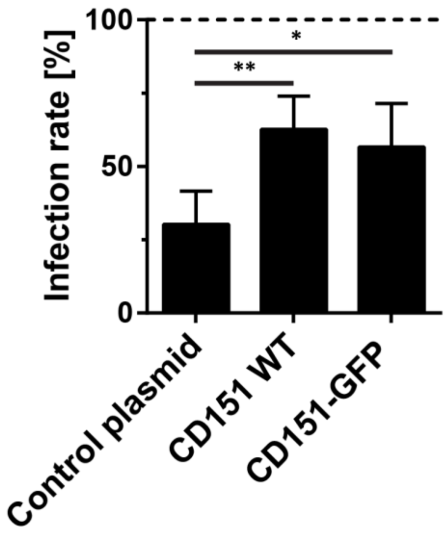

Figure S8. *Rescue of infection by CD151 expression after CD151 knockdown.*
CD151 was knocked down in HeLa cells by siRNA transfection targeting the non-coding 3'UTR region of the endogenous CD151 mRNA. After one day, cells were transfected with a control plasmid or plasmids for expression of non-tagged CD151 (CD151-WT) or GFP-tagged CD151 (CD151-GFP). One day later, cells were incubated with 100 vge per cell. Infection rate was measured 24 hours after PsVs addition in the luciferase based infection assay, normalizing luciferase activity to dehydrogenase activity. Values are given as means ± SD (n = 3 - 4 biological replicates). Statistical analysis was performed employing the unpaired Student's t-test (*, $p < 0.05$; **, $p < 0,01$).

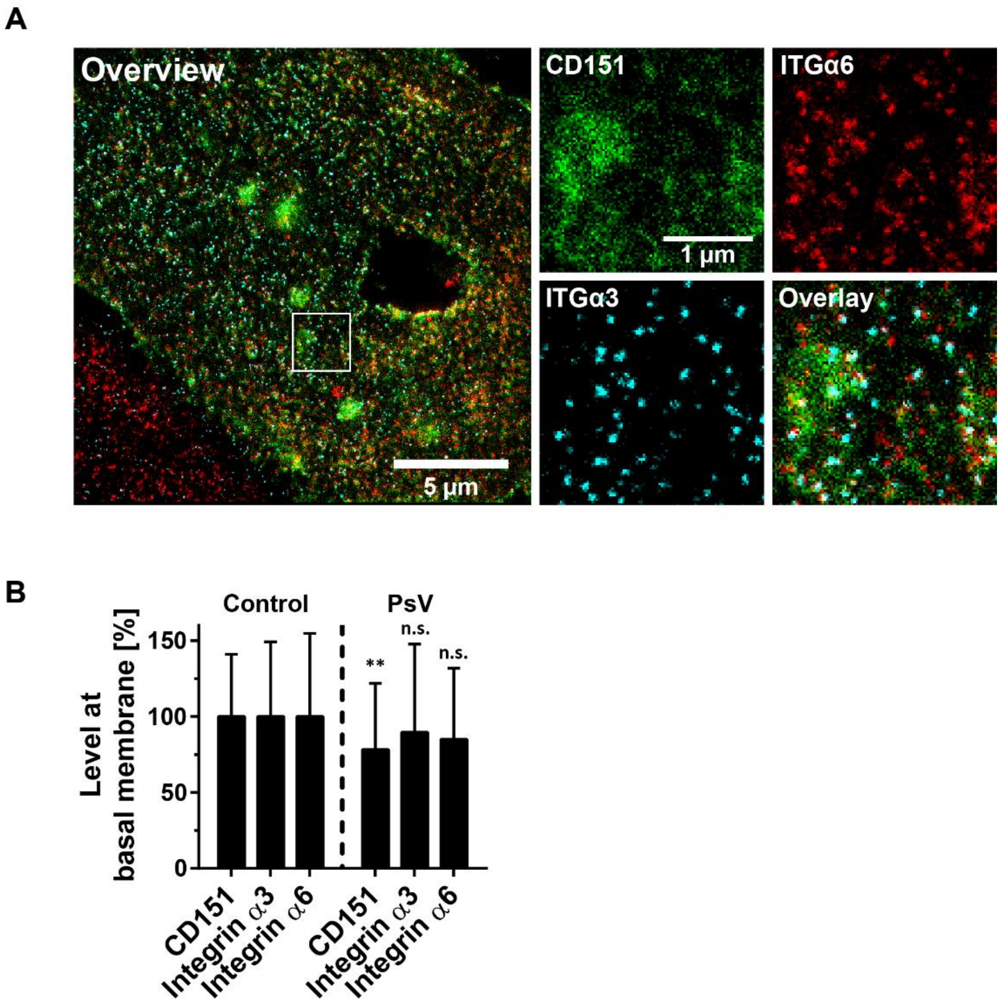

Figure S9. *PsVs induce CD151 patching.*
Experiment from Figure 3, showing the condition with PsVs. (A) Large panel, channel overlay showing a membrane sheet generated from a cell incubated with PsVs. Several CD151 patches are visible that are less frequent in the absence of PsVs (compare Figure 3A). The white box marks an area from which magnified views from the individual channels are shown. Images are displayed with the same lookup tables and scalings as in Figure 3. (B) Mean staining intensities of CD151-GFP, integrin α3 and integrin α6 quantified from membrane sheets generated from cells previously incubated for 5 h without (control) or with PsVs. Values are given as means ± SD (n = 60 membrane sheets collected from three biological replicates). Unpaired Student's t-tests compare control to PsV (**, $p < 0.01$).

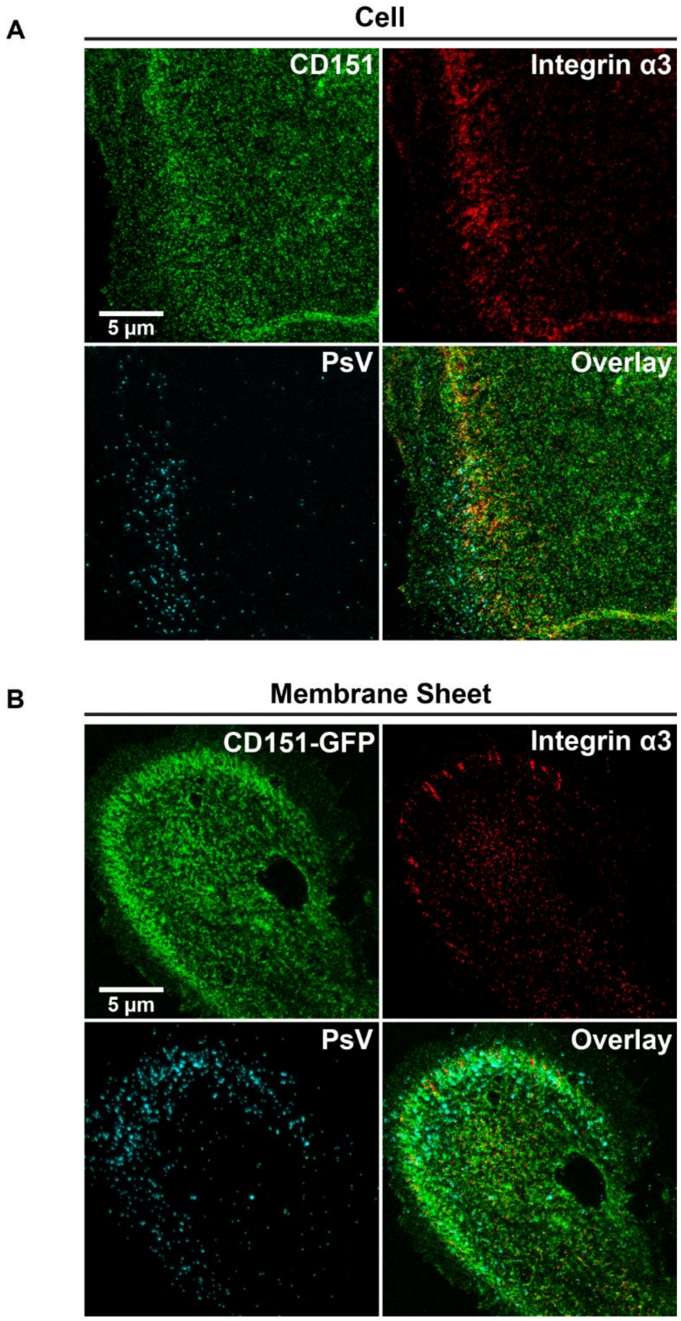

Figure S10. *PsV binding pattern imaged on intact cells or membrane sheets from cells overexpressing CD151-GFP.*
(A) Basal membrane of a HaCaT cell fixed with methanol (from the experiment shown in Figure 4D - F). (B) Membrane sheet generated from a HaCaT cell overexpressing CD151-GFP (from Figure S11). Example images illustrate that independent from the fixation protocol and sample preparation in many cases integrin α3 (linear red lookup table) enriches at the cell periphery, occasionally in elongated structures (B). In particular viral particles (linear cyan lookup table), but also CD151 (linear green lookup table), enrich at the cell periphery. Images are displayed at arbitrary intensity scalings.

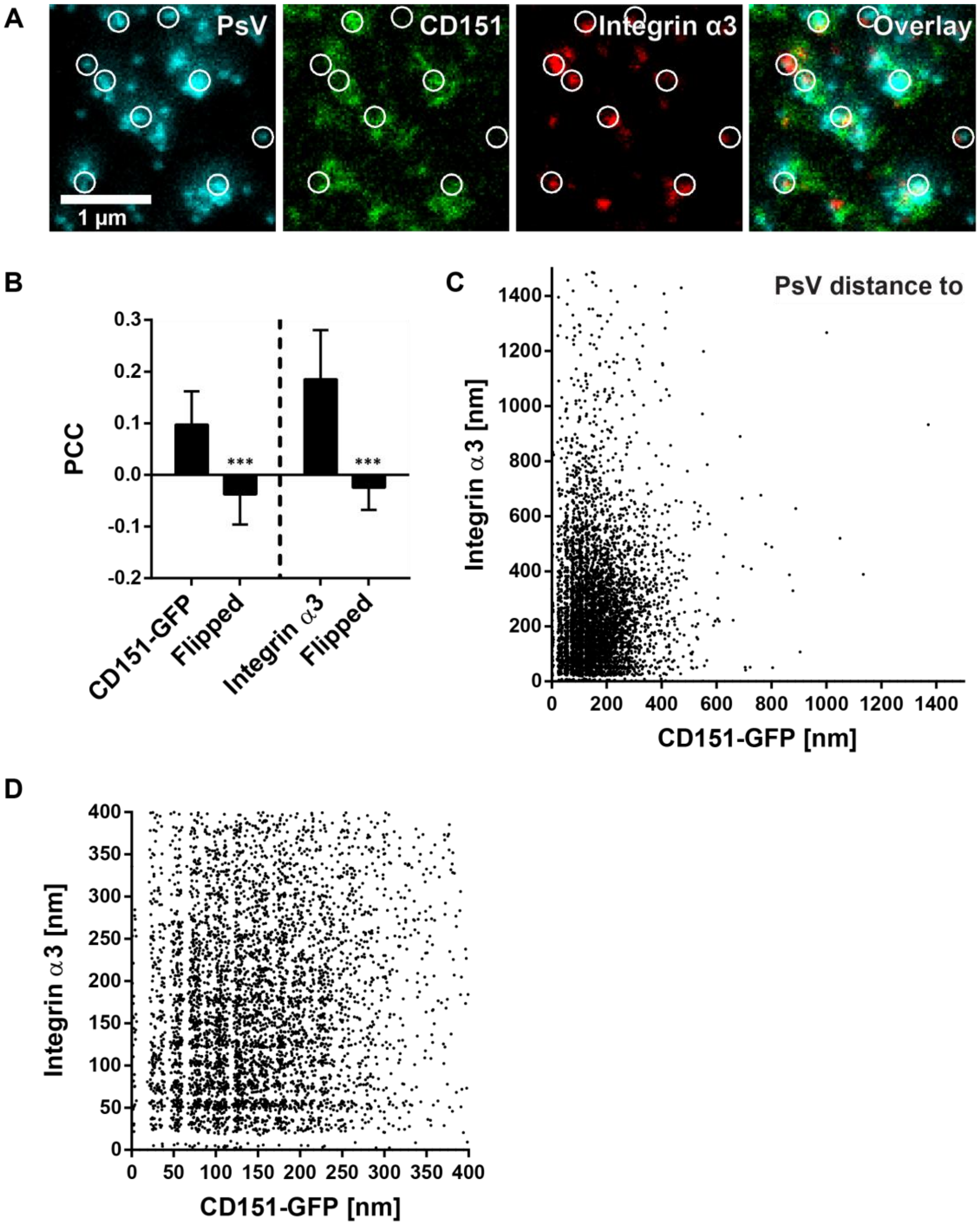

Figure S11. *Viral particle distances to CD151-GFP and integrin α3 maxima.*
HaCaT cells expressing CD151-GFP were incubated with PsVs for 5 h and membrane sheets were generated, fixed, permeabilized, and fluorescently labelled. PsVs (cyan) and integrin α3 (red) were immunostained and CD151-GFP (green) was enhanced with GFP nanobodies. (A) Images from the three channels and an overlay are shown. Images are displayed at arbitrary intensity scalings (linear lookup tables). Circles mark identical pixel locations. (B) Pearson correlation coefficient with the respective control values ('flipped') between PsVs and CD151-GFP (left) and integrin α3 (right). (C) PsV distances to the nearest CD151-GFP and integrin α3 maxima. The analysis includes 6090 PsVs pooled from 60 analysed membrane sheets collected from three biological replicates. Statistical analysis was performed employing the unpaired Student's t-test comparing the original to the flipped images (***, $p < 0.001$). (D) Magnified view from (C).

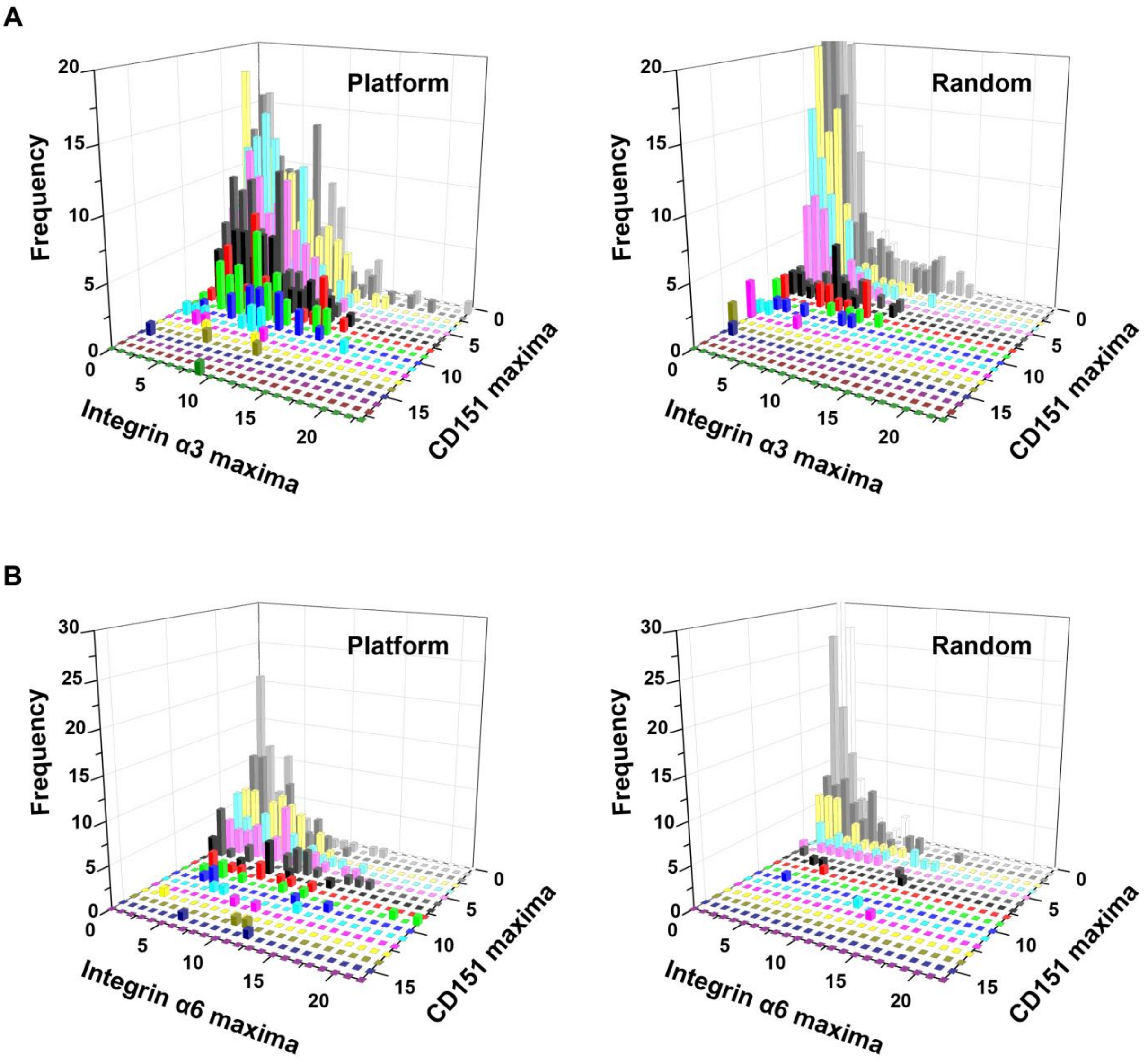

Figure S12. *Histograms illustrating the variability of the number of clusters in the platform area.* Individual maxima/µm^2 from Figure 5C and D plotted as 2D histograms. For each presumed viral particle attachment site, the number of bright integrin α3 (A; 678 PsVs) or integrin α6 (B; 283 PsVs) maxima is plotted versus the number of bright CD151 maxima. Plots are shown for the platform and the randomized area. Please note that the histograms of the randomized areas are truncated at low count bins. Their values at the zero/zero count bins reach 139 for (A) and 68 for (B).

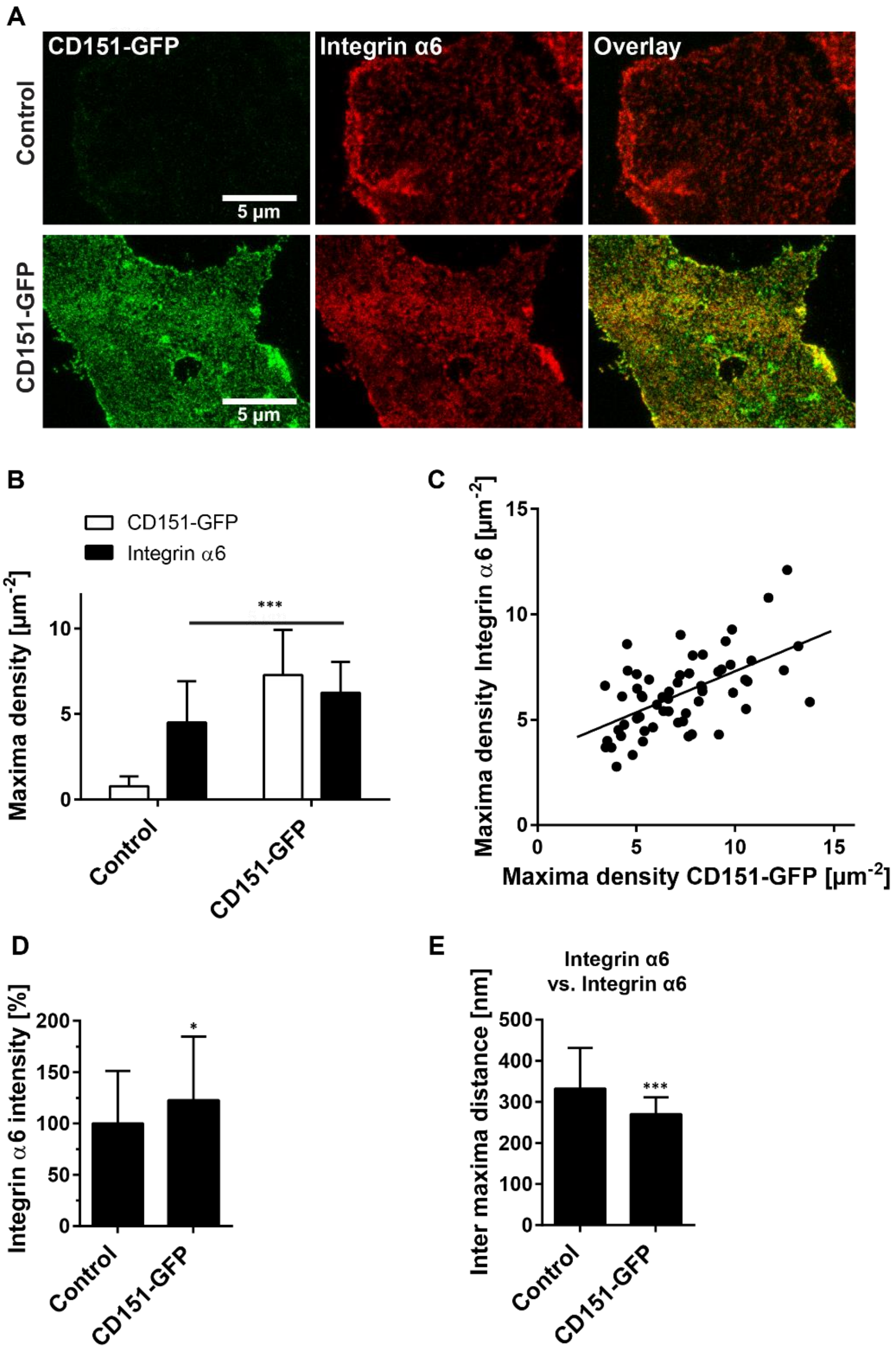

Figure S13. *CD151-GFP overexpression increases the level of cell surface integrin α6.*
(A) Membrane sheets from control and CD151-GFP overexpressing HaCaT cells, stained with antibodies for GFP (green) and integrin α6 (red). Images are displayed at arbitrary intensity scalings (linear lookup tables). (B) From ROIs, the maxima per µm² of the two maxima types was determined, revealing that overexpression of CD151-GFP increases the number of integrin α6 maxima. Please note that the detected CD151-GFP maxima under non-overexpression conditions result from background signal. (C) Plotting from individual membrane sheets the density of integrin α6 against the CD151-GFP maxima suggests a trend towards more integrin α6 maxima with increasing CD151-GFP expression level. A regression line was fitted to the data ($R^2 = 0.3296$). (D) Average integrin α6 staining and (E) inter-maxima distances in control and overexpression condition. The distance between integrin maxima is reduced upon CD151-GFP overexpression due to the enhanced integrin maxima density. Values are shown as means ± SD (n = 59 membrane sheets collected from three biological replicates). The unpaired Student's t-test compares control to overexpression (***, $p < 0.001$; *, $p < 0.05$).

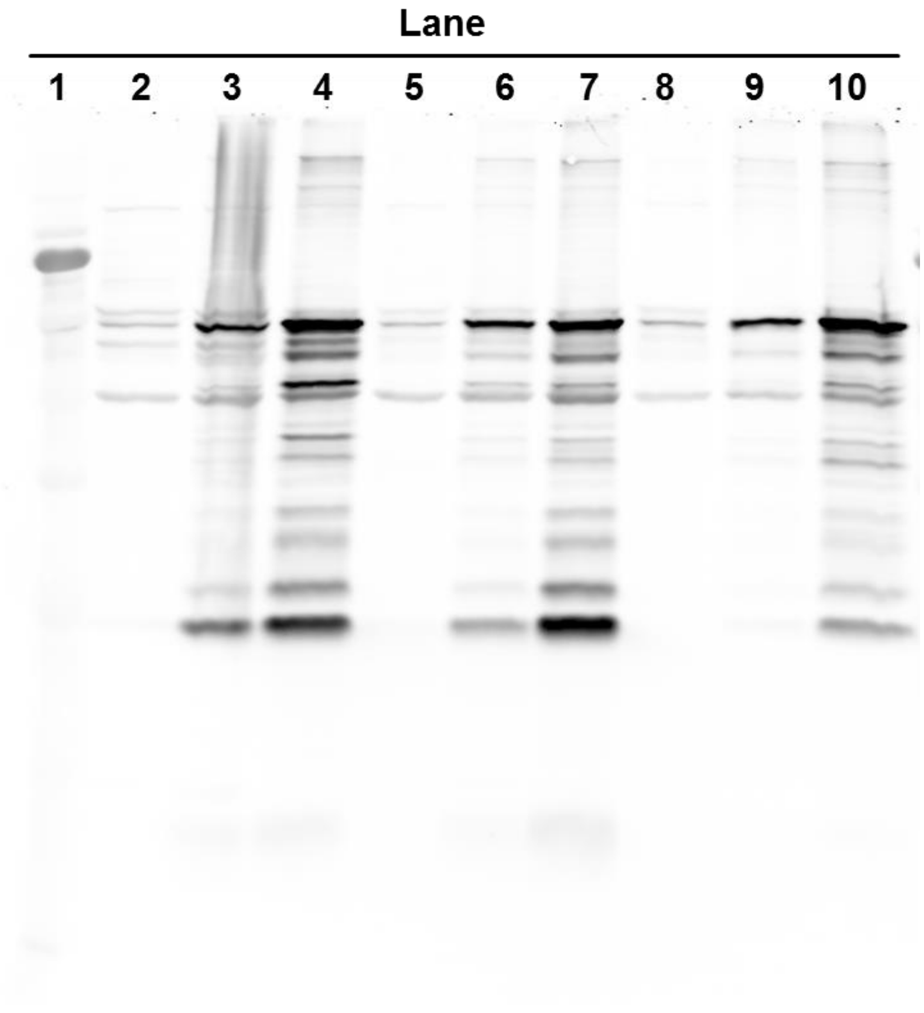

Figure S14. *Full Western blot for L1 from Figure 1.*
Lanes cropped and used for Figure 1 are lane 3 (Control siRNA), lane 9 (Integrin α3 siRNA) and lane 6 (Integrin α6 siRNA).

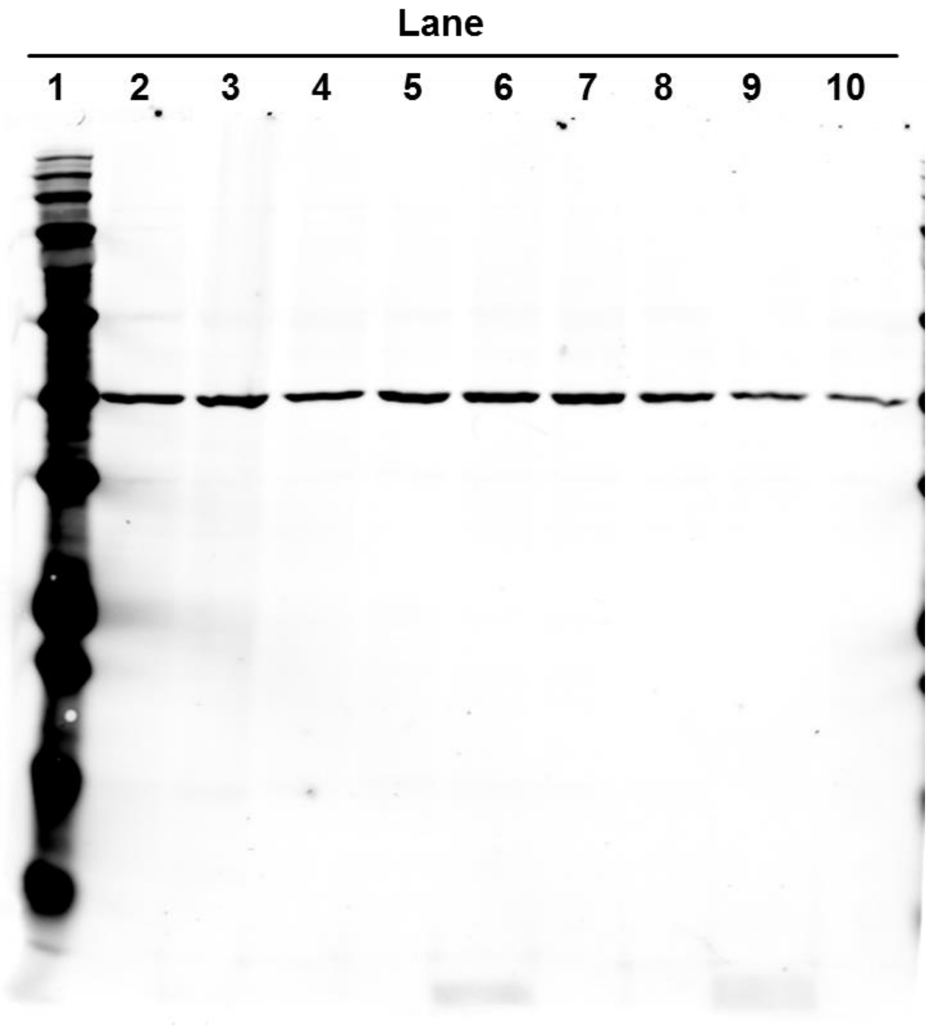

Figure S15. *Full Western blot for actin from Figure 1.*
Lanes cropped and used for Figure 1 are lane 3 (Control siRNA), lane 9 (Integrin α3 siRNA) and lane 6 (Integrin α6 siRNA).

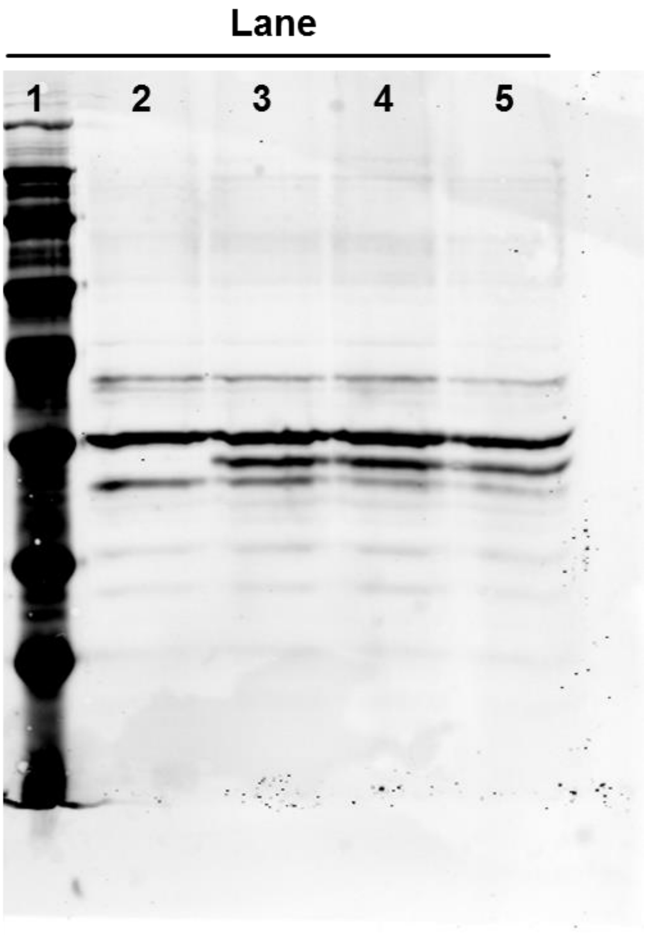

Figure S16. *Full Western blot for L1 from Figure 2.*

.

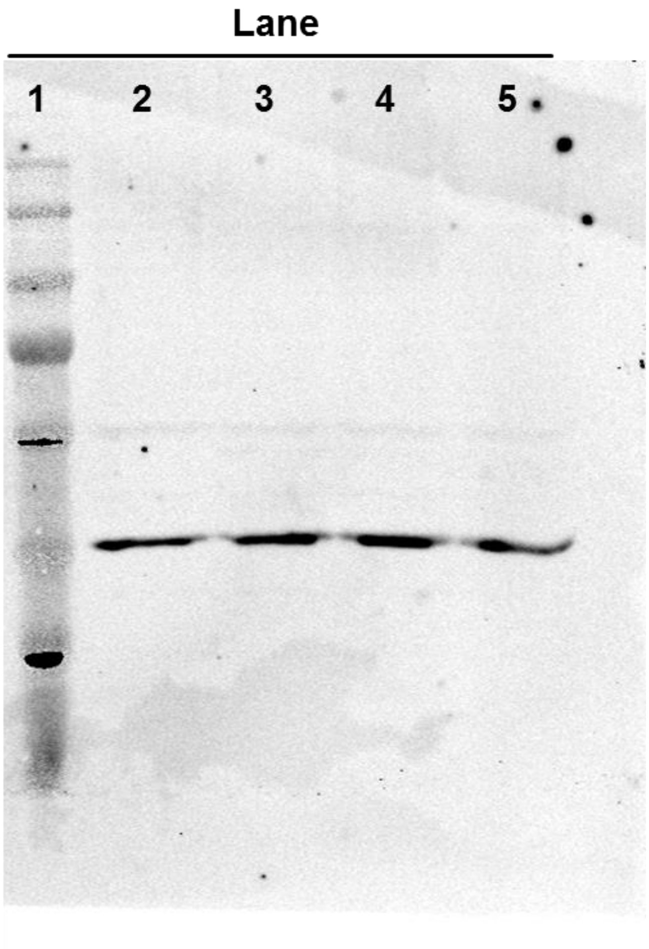

Figure S17. *Full Western blot for actin from Figure 2.*

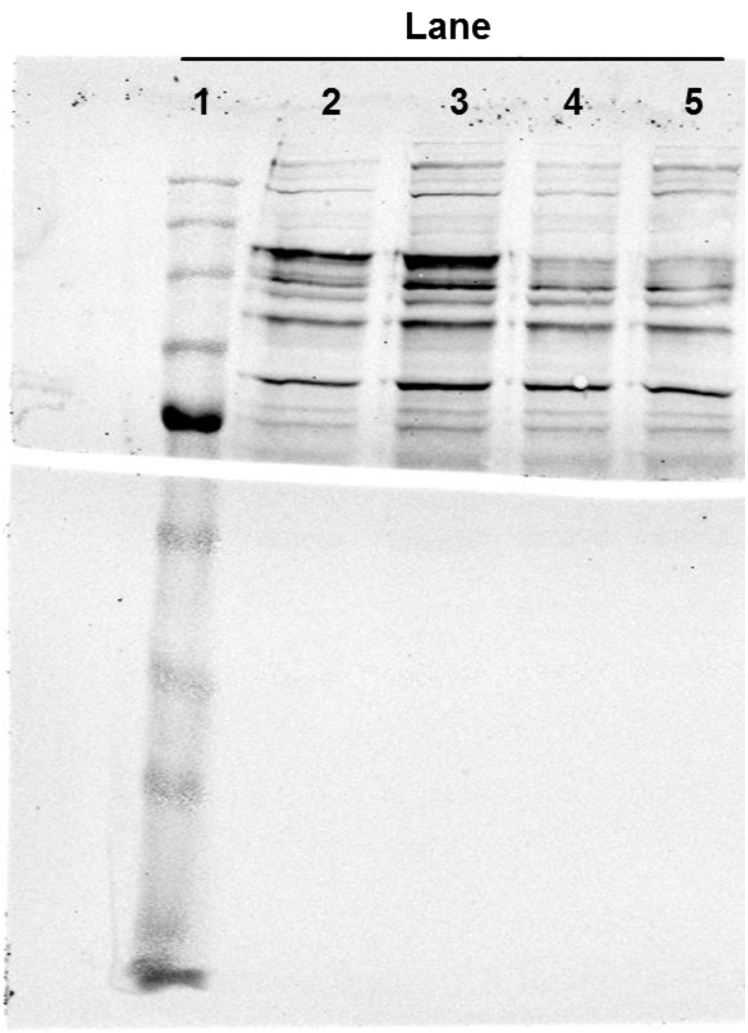

Figure S18. *Full Western blot for integrin α6 from Figure S1A.*
The blot was cut in two parts for staining of the upper part for integrin α6 and the lower part for actin (Figure S19; both antibodies raised in rabbits).

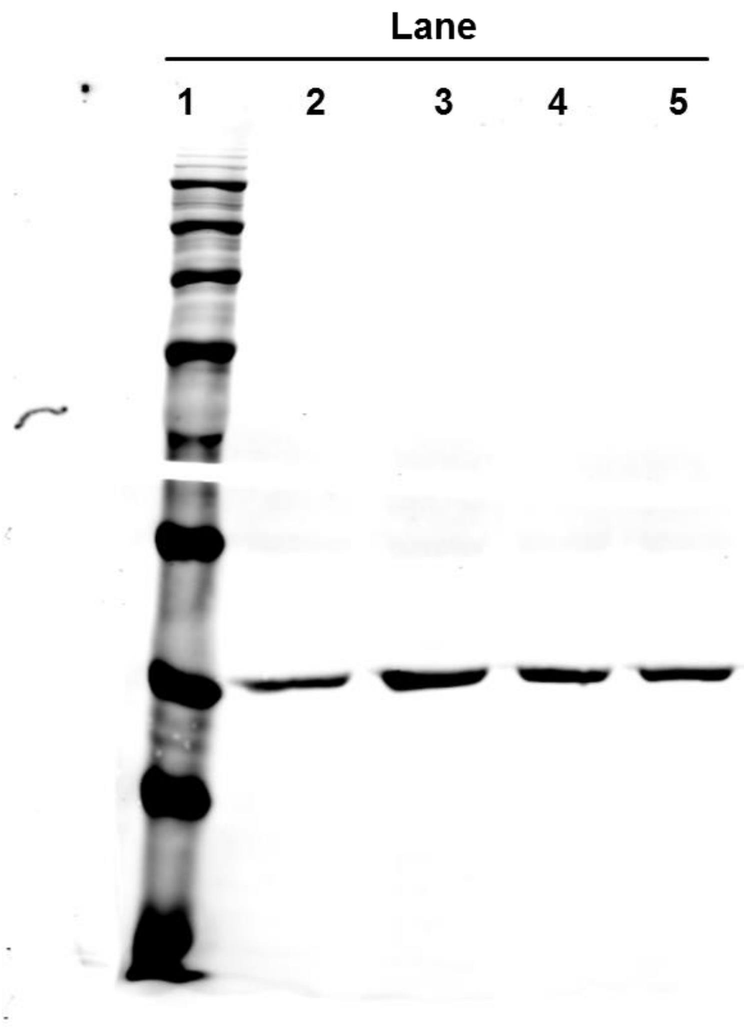

Figure S19. *Full Western blot for actin from Figure S1A.*
The blot was cut in two parts for staining of the upper part for integrin α6 (Figure S18) and the lower part for actin (both antibodies raised in rabbits).

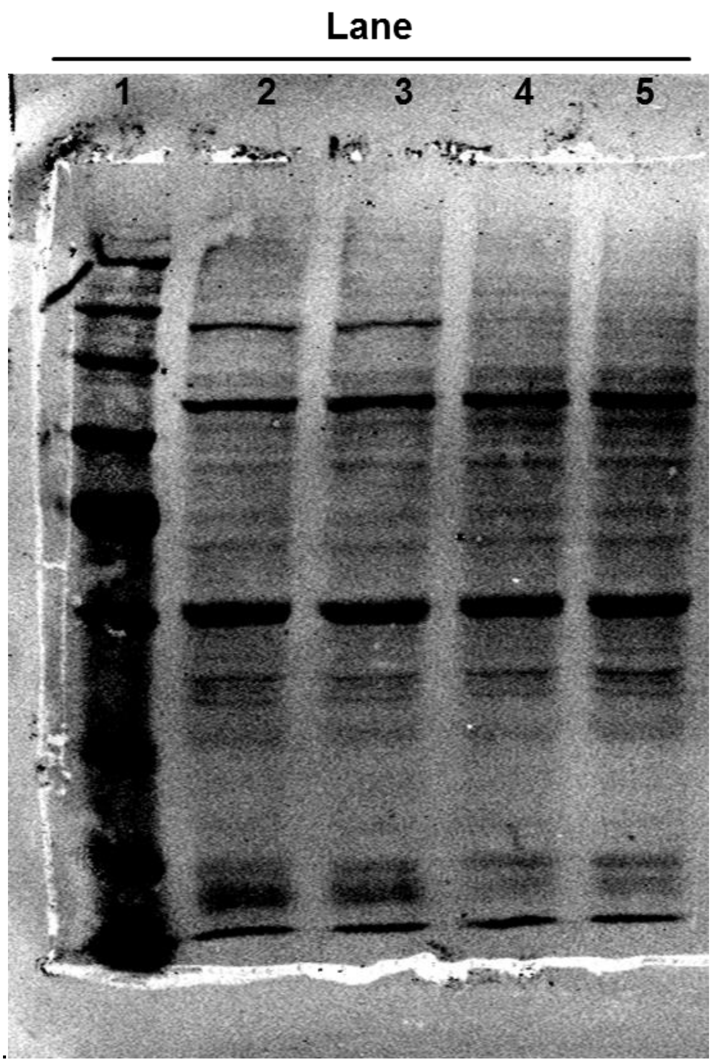

Figure S20. *Full Western blot for integrin α3 from Figure S1B.*

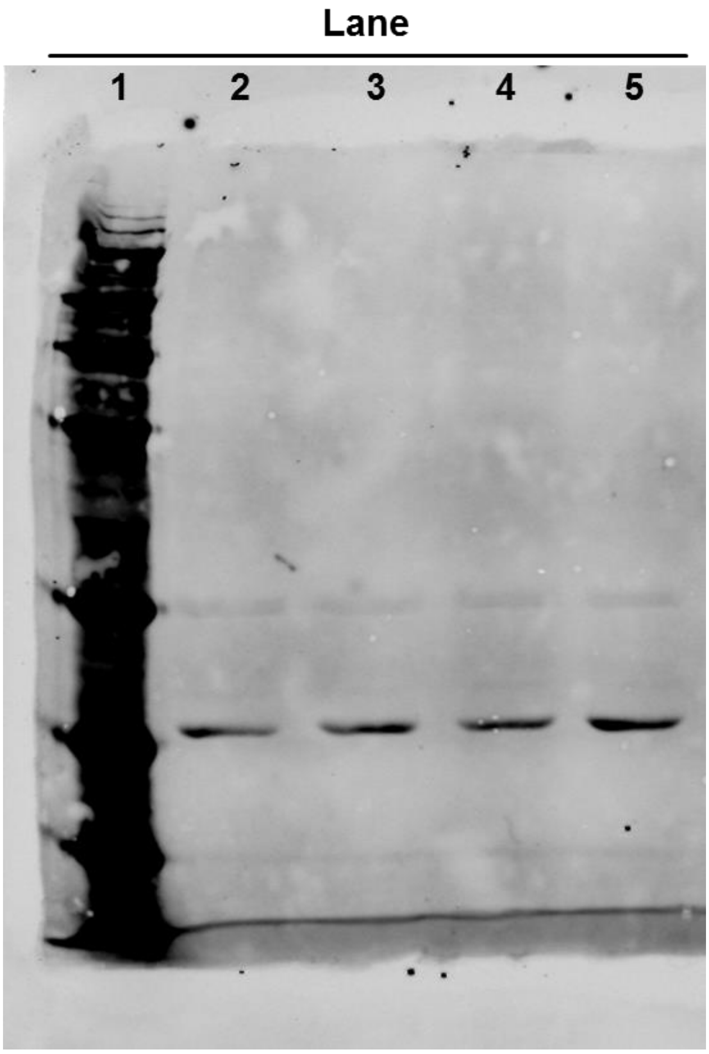

Figure S21. *Full Western blot for actin from Figure S1B.*

Supplementary References

1. Baker, T. S. *et al.* Structures of bovine and human papillomaviruses. Analysis by cryoelectron microscopy and three-dimensional image reconstruction. *Biophysical journal* **60,** 1445–1456 (1991).

2. Aquino, D. *et al.* Two-color nanoscopy of three-dimensional volumes by 4Pi detection of stochastically switched fluorophores. *Nature methods* **8,** 353–359; 10.1038/nmeth.1583 (2011).

3. Sieber, J. J., Willig, K. I., Heintzmann, R., Hell, S. W. & Lang, T. The SNARE Motif Is Essential for the Formation of Syntaxin Clusters in the Plasma Membrane. *Biophysical journal* **90,** 2843–2851; 10.1529/biophysj.105.079574 (2006).
